# Hypoglycemia-Exacerbated Mitochondrial Connexin 43 Accumulation Aggravates Cardiac Dysfunction in Diabetic Cardiomyopathy

**DOI:** 10.3389/fcvm.2022.800185

**Published:** 2022-03-16

**Authors:** Xing Wei, Andrew Chia Hao Chang, Haishuang Chang, Shan Xu, Yilin Xue, Yuanxin Zhang, Ming Lei, Alex Chia Yu Chang, Qingyong Zhang

**Affiliations:** ^1^Department of Cardiology, Ninth People's Hospital, Shanghai Jiao Tong University School of Medicine, Shanghai, China; ^2^Shanghai Institute of Precision Medicine, Ninth People's Hospital, Shanghai Jiao Tong University School of Medicine, Shanghai, China

**Keywords:** hypoglycemia, diabetic cardiomyopathy, mitochondrial, connexin 43, Src

## Abstract

**Background:**

Diabetic cardiomyopathy (DCM) is a complex multifaceted disease responsible for elevated heart failure (HF) morbidity and mortality in patients with diabetes mellitus (DM). Patients with DCM exhibit subclinical diastolic dysfunction, progression toward systolic impairment, and abnormal electrophysiology. Hypoglycemia events that occur spontaneously or due to excess insulin administration threaten the lives of patients with DM—with the increased risk of sudden death. However, the molecular underpinnings of this fatal disease remain to be elucidated.

**Methods and Results:**

Here, we used the established streptozotocin-induced DCM murine model to investigate how hypoglycemia aggravates DCM progression. We confirmed connexin 43 (Cx43) dissociation from cell–cell interaction and accumulation at mitochondrial inner membrane both in the cardiomyocytes of patients with DM and DCM murine. Here, we observed that cardiac diastolic function, induced by chronic hyperglycemia, was further aggravated upon hypoglycemia challenge. Similar contractile defects were recapitulated using neonatal mouse ventricular myocytes (NMVMs) under glucose fluctuation challenges. Using immunoprecipitation mass spectrometry, we identified and validated that hypoglycemia challenge activates the mitogen-activated protein kinase kinase (MAPK kinase) (MEK)/extracellular regulated protein kinase (ERK) and inhibits phosphoinositide 3-kinase (PI3K)/Akt pathways, which results in Cx43 phosphorylation by Src protein and translocation to mitochondria in cardiomyocytes. To determine causality, we overexpressed a mitochondrial targeting Cx43 (mtCx43) using adeno-associated virus serotype 2 (AAV2)/9. At normal blood glucose levels, mtCx43 overexpression recapitulated cardiac diastolic dysfunction as well as aberrant electrophysiology *in vivo*. Our findings give support for therapeutic targeting of MEK/ERK/Src and PI3K/Akt/Src pathways to prevent mtCx43-driven DCM.

**Conclusion:**

DCM presents compensatory adaptation of mild mtCx43 accumulation, yet acute hypoglycemia challenges result in further accumulation of mtCx43 through the MEK/ERK/Src and PI3K/Akt/Src pathways. We provide evidence that Cx43 mislocalization is present in hearts of patients with DM hearts, STZ-induced DCM murine model, and glucose fluctuation challenged NMVMs. Mechanistically, we demonstrated that mtCx43 is responsible for inducing aberrant contraction and disrupts electrophysiology in cardiomyocytes and our results support targeting of mtCx43 in treating DCM.

## Introduction

The International Diabetes Federation (IDF) Diabetes Atlas 2019 estimated the number of people living with diabetes mellitus (DM) would reach 693 million by 2045, representing 9.3% of the global adult population (20-79 years) (1). Myocardial dysfunction that progresses to heart failure is present in patients with DM and is clinically defined as diabetic cardiomyopathy (DCM) (2). DCM hearts are characterized by cardiac remodeling, early onset of diastolic dysfunction followed by systolic impairment, and eventually progresses to heart failure with reduced ejection fraction (HFrEF) (3). Compared to coronary artery disease-induced dilated cardiomyopathy, DCM has worse prognosis (4, 5). Compared to other cardiomyopathies, DCM exhibits metabolic dysfunction, electrophysiology, and insulin resistance (6, 7)—making DCM management much more challenging. Moreover, our understanding of the molecular underpinnings of DCM remains limited.

Hypoglycemia is a major challenge in DM management (8) that significantly increases the mortality rate in both the type 1 and type 2 patients with DM (9, 10). Hypoglycemia, defined as blood glucose ≤ 3.0 mM, can result in cognitive confusion, loss of consciousness, seizures, and even death in both the young and elderly patients with DM (11–14). Prolonged hypoglycemia-induced neuroglycopenia is rare; most fatal hypoglycemic episodes result in cardiac dysfunction, especially ventricular arrhythmias (12, 15). Experimentally-induced hypoglycemic events in patients with type 1 or type 2 DM resulted in proarrhythmogenic cardiac repolarization with prolonged QT intervals (16, 17). In rodent models, severe hypoglycemia (blood glucose < 1.0 mM) leads to prolonged QT interval, ventricular ectopy, and high-degree atrioventricular blockage (18). How hypoglycemia can induce cardiac dysfunction and abnormal electrophysiology remains to be elucidated.

Gap junctions (GJs) are channels that directly connect two adjacent cells with two connexin hemichannels, allowing passage of ions (Na^+^, K^+^, and Ca^2+^) and proteins and molecules <1.5 kDa (19) and are tightly regulated in response to intracellular and extracellular signals (20, 21). Impaired gap junction-mediated arrhythmias are prevalent among patients with acute myocardial infarction and heart failure (22, 23). Connexin 43 (Cx43), compared to other connexin proteins, is expressed highest in the heart and constitutes the main component of ventricular GJs (24). Intercellular communication between cardiomyocytes through Cx43 allows for rapid electrical signal diffusion and synchronous ventricular contraction. Myocardial Cx43 is trafficked via three main pathways: (i) autophagosomal degradation via direct fusion with lysosomes (25) or phagosomes (26, 27); (ii) lateralization to the lateral membrane; and (iii) translocation to the inner mitochondrial membrane (IMM) through heat shock protein (HSP) 90-dependent translocase (28). Previously, we have demonstrated that hypoglycemic challenge resulted in decreased Cx43 expression in hyperglycemia-cultured H9c2 cells (29), suggestive an active role of Cx43 in DCM progression.

In this study, we first observed an increase co-localization in Cx43 and mitochondria in cardiac tissue sections of diabetic patients. Then, we used streptozotocin (STZ)-induced DCM murine model to investigate the role of Cx43 trafficking due to chronic hyperglycemia and acute hypoglycemia challenge. STZ animals exhibit diastolic dysfunction and showed an increase arrhythmia susceptibility and Cx43 translocation to mitochondria IMM upon insulin-induced hypoglycemic challenge. Using neonatal mouse ventricular myocytes (NMVMs), we show that glucose fluctuations can indeed result in contractile dysfunction and recapitulate Cx43 translocation to mitochondria. Molecularly, we demonstrate that activation of MEK/ERK/Src and inhibition of PI3K/Akt/Src pathways are responsible for mitochondrial Cx43 (mtCx43) accumulation. Overexpression of mtCx43 is sufficient to recapitulate hypoglycemia-aggravated cardiac dysfunction and abnormal electrophysiology both *in vitro* and *in vivo*. Together, this study provides mechanistic insight into hypoglycemia-aggravated DCM with potential new avenues for therapeutic designs.

## Materials and Methods

### Ethics

For the collection and use of human cardiac tissue, an informed consent was obtained from subjects and all the protocols were reviewed and approved by the Ethics Review Committee at the Ninth People's Hospital, Shanghai Jiao Tong University School of Medicine, China (SH9H-2020-TK238-1). All the procedures performed in these studies were in accordance with the 1964 Helsinki Declaration and its later amendments or comparable ethical standards. All the participants gave a written informed consent. The general clinical characteristics of the patients are given in Supplementary Table S1.

For animal use, all the protocols were approved by the Laboratory Animal Care Ethics Review Committee at the Ninth People's Hospital, Shanghai Jiao Tong University School of Medicine, China (SH9H-2020-A234-1). For adult mice, animals were euthanized by deep isoflurane (5%) and sacrificed by cervical dislocation. For neonatal mice (within 72 h) that are resistant to CO_2_, animals were euthanized by decapitation.

### Streptozotocin to DCM Mouse Model and Treatment

C57BL/6N mice (JSJ Lab, Shanghai, China) were acquired and used to generate type 1 DM disease model in both the male and female mice. The exact number of animals for individual experiments is reported in figure legends. Type 1 DM disease model was established using intraperitoneal injection of STZ (150 mg/kg body weight, V900890, Sigma-Aldrich, USA) after a 10-h fast in 8 weeks old C57BL6/N mice. In parallel, equal volume of 0.1 M sodium citrate was injected and served as control. Blood glucose measurements were monitored using an Accu-Chek Active Blood Glucose Meter (Roche Diabetes Care GmbH, Mannheim, Germany) once every 2 weeks post-STZ induction. Three consecutive random blood glucose measurements > 16.7 mM deemed modeling success and were used as type 1 DM model (DM group) in subsequent analyses. STZ mice post-10 weeks of initial injection exhibiting stable diastolic dysfunction (defined as the DCM group) were subjected to hypoglycemic challenge. Insulin (100 IU, Yuanye Biotechnology Corporation Ltd., Shanghai, China) was injected intraperitoneally to induce hypoglycemia (HDCM group), <3.0 mmol/l glucose postinsulin induction, and animals were sacrificed 120 min postinjection (**Figure 2A**).

### Echocardiography and ECG

Before the experiments, the animals were sedated in a chamber with 3% isoflurane in a mixture of medical air (flow rate: 0.2 L/min) and O_2_ (flow rate: 0.2 L/min). During the measurements, the anesthesia was maintained at 1–2% isoflurane through a customized anesthesia mask. Temperature was maintained at 36–37°C with a heating pad. Echocardiography was performed using a MX400 ultra-high frequency linear array transducer (18–38 MHz, center transmit: 30 MHz, and axial resolution: 50 μm) on the Vevo 3100 high-resolution Imaging System (FUJIFILM VisualSonics, Toronto, Canada). Determination of cardiac systolic function, ejection fraction (EF), and fractional shortening (FS) was performed in a semiautomatic manner in M-mode (long-axis view). Doppler imaging was used to measure early to late ventricular filling velocity (E/A) ratio and early mitral inflow velocity to early diastolic mitral annulus velocity (E/E') ratio. Echocardiography measurements were recorded twice a week post-STZ treatment to monitor cardiac function. Echocardiographic analysis was performed using VevoLAB version 3.0 software package (FUJIFILM VisualSonics, Toronto, Canada). ECG was recorded by the standard limb lead II in anesthetized mice for 5 min at a 10-kHz acquisition frequency. The signal was digitalized using PowerLab physiology recorder (AD Instruments, Sydney, Australia) and analyzed with LabChart 7.0 software program (AD Instruments, Sydney, Australia). The following ECG parameters were analyzed: QTc, QT interval, QRS interval, and JT interval.

### Neonatal Cardiomyocyte Isolation and Treatment

Neonatal mouse ventricular myocytes were isolated from day 1–3 mouse hearts using 0.25% trypsin-ethylenediaminetetraacetic acid (EDTA) (25200072, Gibco, USA). Isolated NMVMs were plated at a seeding density of 1 × 10^4^ cells/cm^2^ in complete Dulbecco's Modified Eagle Medium (DMEM) supplemented with 10% fetal bovine serum (FBS) and cultured at 37°C and 5% CO_2_ for 3 days. Once NMVMs start to beat, cells were treated with 10% FBS DMEM containing either 5.5 mM glucose concentrations with 27.8 mM mannitol for 4 h [normal glucose group (NG)], 5.5 mM glucose concentrations with 27.8 mM mannitol for 2 h followed by 2.5 mM glucose supplemented with 30.8 mM mannitol for 2 h [normal glucose following low glucose group (NLG)], 33.3 mM glucose concentrations for 4 h [high glucose group (HG)], or 33.3 mM glucose for 2 h followed by 2.5 mM glucose supplemented with 30.8 mM mannitol for 2 h [high glucose following low glucose group (HLG)] (**Figure 3A**). MEK1/2 inhibitor U0126 (10 μM, S1901, Beyotime, Shanghai, China), ERK1/2 activator ceramide C6 (10 μM, 860506P, Avanti Polar Lipids, Alabaster, USA), Akt inhibitor triciribine (15 μM, SF2721, Beyotime, Shanghai, China), and Akt activator Sc79 (10 μM, SF2730, Beyotime, Shanghai, China) were added in HLG-treated NMVMs (**Figure 7A**).

### Impedance (IMP) and Extracellular Field Potential (EFP) Measurement

The IMP (indicating contractility) and EFP (indicating surface voltage) of NMVMs were measured using the CardioExcyte 96 (Nanion Technologies, Munich, Germany). Cells (4 × 10^4^ to 6 × 10^4^ cells/well) were seeded in each well using DMEM/10% FBS on an NSP-96 plate and cultured for 2 days prior to the assay. Contractile function of spontaneous beating NMVMs was measured using the CardioExcyte Control software (Nanion Technologies, Germany). The 30 s video recordings of IMP and EFP were taken every 10 min for 4 h. Data were analyzed using the DataControl 96 (Nanion Technologies, Germany) and data statistics were compared with 0 h (NG condition) for normalization.

### Immunoelectron Microscopy

A small piece of heart sample was obtained by a fine-needle biopsy and placed in a type A (200 μm depth) specimen carrier filled with 1-hexadecen (H2131, Sigma-Aldrich, USA). After being covered with a type B carrier, the sample was snap frozen using a High-pressure Freezing Machine (EM ICE, Wetzlar, Germany) and rapidly transferred into liquid nitrogen for storage. Next, frozen samples were transferred into a Freeze-substitution Unit (EM AFS2, Leica, Germany) for substitution. Samples were incubated for 48 h in acetone (G75902A, Sigma-Aldrich, USA) containing 0.2% uranyl acetate reagent (22400, Electron Microscopy Science, Hatfield, Pennsylvania, USA) at −90°C. The temperature was gradually raised to −50°C for 4 h span, maintained at −50°C for 12 h, gradually warmed up to −30°C for 4 h span, and followed by another −30°C incubation for 2 h. Samples were then washed 3 × 15 min with pure acetone. The samples were then stepwise infiltrated by HM20 resin (14340, Electron Microscopy Science, Switzerland) with grades of 25, 50, 75%, and pure resin (1 h incubation each) at −30°C. After infiltration in pure resin overnight, the samples were embedded in gelatin capsules (CZ130, Zhongjing Technology Corporation Ltd., Beijing, China). The samples were polymerized under UV light on the Leica EM AFS2 machine (Leica, Germany) for 48 h at −30°C and 12 h at 25°C and then trimmed and ultra-thin sectioned with an UC7 Microtome (Leica, Germany). Serial thin sections (100 nm thick) were collected on the Formvar-coated nickel grids.

The Formvar-coated nickel grids with sections were incubated in 0.01 M phosphate-buffered saline (PBS) containing 1% bovine serum albumin (BSA) (B2064, Sigma-Aldrich, USA), 0.05% Triton X-100 (X-100, Sigma-Aldrich, USA), and 0.05% Tween 20 (P1379, Sigma-Aldrich, USA) for 5 min. The sections were then incubated in rabbit anti-GJA1 primary antibody diluted in 0.01 M PBS contain 1% BSA and 0.05% Tween 20 (1:100) at 4°C overnight. Sections were washed twice for 6 min with 0.01 M PBS; the sections were incubated in goat antirabbit conjugated with 10 nm gold secondary antibody (1:50, G7402, Sigma-Aldrich, USA) diluted in 0.01 M PBS containing 1% BSA and 0.05% Tween 20 for 1 h at 25°C. Samples were washed twice for 6 min with 0.01 M PBS, twice for 4 min with distilled water, dried at 25°C, and then examined and photographed using transmission electron microscopy (FEI Talos L120C; Thermo Fisher Scientific, Waltham, Massachusetts, USA). Images were analyzed with Imaris software (Oxford Instruments, UK) and Image J software (National Institutes of Health, USA).

### Immunofluorescent Staining

Cells were fixed for 5 min using cold 4% paraformaldehyde (PFA) in PBS. Fixed samples were then washed 3 × 5 min and blocked with blocking buffer (0.3% Triton X-100/20% FBS/PBS) for 1 h at room temperature (RT). Primary antibodies anti-vimentin antibody (rabbit monoclonal, 1:1,000, ab92547, Abcam, Cambridge, UK), anti-ACTN2 (mouse monoclonal, 1:500, ab9465, Abcam, Cambridge, UK), anti-GJA1 antibody (mouse monoclonal, 1:200, ab78055, Abcam, Cambridge, UK), anti-N-cadherin antibody (rabbit polyclonal, 1:500, ab18203, Abcam, Cambridge, UK), and anti-TOM20 (rabbit polyclonal, 1:1,000, 11802-1-AP, Proteintech, Chicago, USA) were diluted in blocking buffer and incubated overnight at 4°C protected from light. Samples were washed 3 × 10 min with blocking buffer and then stained with goat antimouse or antirabbit Alexa 488, 546, or 647 secondary antibodies (1:1,000, Invitrogen, USA) incubation in the dark at RT for 1 h. Samples were washed 3 × 10 min with blocking buffer and 3 × 10 min with PBST (0.3% Triton X-100/PBS) and stained with 4',6-diamidino-2-phenylindole (DAPI) for 5 min at RT. The samples were then washed 3 × 10 min and mounted with antifade mounting medium (E675011; BBI Life Science, Shanghai, China). All cell immunofluorescence was performed using an LSM 880 confocal microscope (Zeiss, Oberkochen, Germany). Multiple layers of detailed three-dimensional images were superimposed and analyzed using the Imaris software (Oxford Instruments, Abingdon, UK). Staining intensity values were quantified and plotted as mean ± SEM. The Pearson's correlation coefficient in the colocalized volume was calculated to analyze the colocalization correlation between proteins.

### Coimmunoprecipitation (Co-IP)

Cell lysates were extracted from treated cells using RIPA Lysis Buffer (P0013B; Beyotime, Shanghai, China) supplemented with protease and phosphatase inhibitor cocktail (P1045; Beyotime, Shanghai, China). Next, a bicinchoninic acid (BCA) assay (P0012; Beyotime, Shanghai, China) was performed to assess protein concentrations at 300 μg/ml and incubated with primary antibodies in dilution buffer (P0256; Beyotime, Shanghai, China) overnight at 4°C. The complexes were mixed with Protein G Agarose (10001D; Invitrogen, USA) and shaken for 3 h at 4°C to capture the antigen–antibody mixture. The beads were then washed five times with cell lysis buffer [20 mM Tris-HCl, 150 mM glycerol, 0.5% Triton X-100, 1 mM EDTA, and 1 mM ethylene glycol tetraacetic acid (EGTA)]. The eluted proteins were analyzed using the Q Exactive HF Orbitrap Mass Spectrometer (Thermo Fisher Scientific). Data were analyzed using DAVID Bioinformatics Resources version 6.8 (https://david.ncifcrf.gov/).

### Immunoblotting

Cells were lysed using RIPA Lysis Buffer (P0013B; Beyotime, Shanghai, China) supplemented with protease and phosphatase inhibitor cocktail (P1045; Beyotime, Shanghai, China) for 30 min and then centrifuged at 15,000 g for 15 min at 4°C. Protein concentrations were determined using a BCA assay (P0012; Beyotime, Shanghai, China). Samples were mixed with 5X sodium dodecyl sulfate (SDS) loading buffer (P0285; Beyotime, Shanghai, China), boiled for 5 min, and chilled on ice for 5 min before loading. Proteins (30 μg) were loaded onto a 10% polyacrylamide gel electrophoresis (PAGE) Bis-Tris gel (PG112; Epizyme, Cambridge, Massachusetts, USA) for electrophoresis, then transferred onto polyvinylidene difluoride (PVDF) membranes, and blocked with blocking buffer (P0252; Beyotime, Shanghai, China). Primary antibodies anti-GJA1 antibody (mouse monoclonal, 1:200, ab78055, Abcam, Cambridge, UK), anti-p-Cx43 antibody (rabbit monoclonal, 1:1,000, 52559, CST, Boston, USA), anti-Src (rabbit monoclonal, 1:500, 11097-1-AP, Proteintech, Chicago, USA), anti-p-Akt antibody (mouse monoclonal, 1:500, 66444-1-lg, Proteintech, Chicago, USA), anti-p-ERK1/2 (rabbit polyclonal, 1:1000, 28733-1-AP, Proteintech, Chicago, USA), and anti-glyceraldehyde-3-phosphate dehydrogenase (GAPDH) antibody (mouse monoclonal, 1:500, 60004-1-lg, Proteintech, Chicago, USA) were diluted in dilution buffer (P0256; Beyotime, Shanghai, China) and added to PVDF membranes overnight at 4°C. Blots were washed and probed with horseradish peroxidase (HRP)-conjugated secondary antibodies for 1 h at 25°C. The blots were imaged using the ChemiDoc Developer System (Bio-Rad, Hercules, California, USA). All the bands were exposed within the linear range to avoid overexposure. Data images were processed and analyzed using ImageJ software version 1.51 (National Institutes of Health, Bethesda, Maryland, USA).

### Cloning of Mitochondria-Targeted EGFP and Cx43 Vectors and Transfection

Connexin 43 coding region was first PCR amplified and inserted into an pAAV2/9-hTNNT2-enhanced Green Fluorescent Protein (EGFP)-mito backbone vector (30) to construct a mtCx43 (**Figure 8A** and Supplementary Figure S5A). The plasmid was amplified and the sequence was verified by Taitool Bioscience Corporation Ltd. (Shanghai, China). pAAV2/9-CMV-GJA1-flag-GFP plasmids were built and verified by Hanbio Biotechnology (Shanghai, China) and used as the conditional control group—the wild-type Cx43 (wtCx43) overexpression group. For overexpression experiments, plasmids were transfected using Lipofectamine 3000 Reagent (L3000001, Invitrogen, USA) following the manufacturer's protocol. NMVMs were seeded in a six-well plate at a density of 5 × 10^5^ cells/well. First, 2 μg of either Cx43-GFP, EGFP-mtCx43, or controls (GFP or mtEGFP) was first mixed with 2 μl P3000 Reagent and 125 μl Lipofectamine 3000 Reagent, diluted with Opti-Minimum Essential Medium (MEM) (1:1), incubated for 10–15 min at 25°C, and added to the culture medium in a drop-wise fashion. Transfected cells were assayed 48 h posttransfection. For overexpression study, C57BL/6N mice were administered with 2E + 11 VG virus through tail vein injection. The animals examined to determine cardiac function at week 2 and sacrificed 4 weeks after infection.

### Statistical Analysis

All the data were analyzed using GraphPad Prism version 8.0 (GraphPad Software, La Jolla, California, USA) and presented as mean ± SEM. For statistical analysis, one-way ANOVA with Tukey's correction for *post-hoc* comparisons and the two-tailed Students *t*-test were used. Statistical significance was set at *P* < 0.05. For complete statistical analyses, please see Supplementary Excel.

## Results

### Abnormal Cx43 Expression and Localization in Myocardial Tissue of Patients With DM

**Figure 1:**
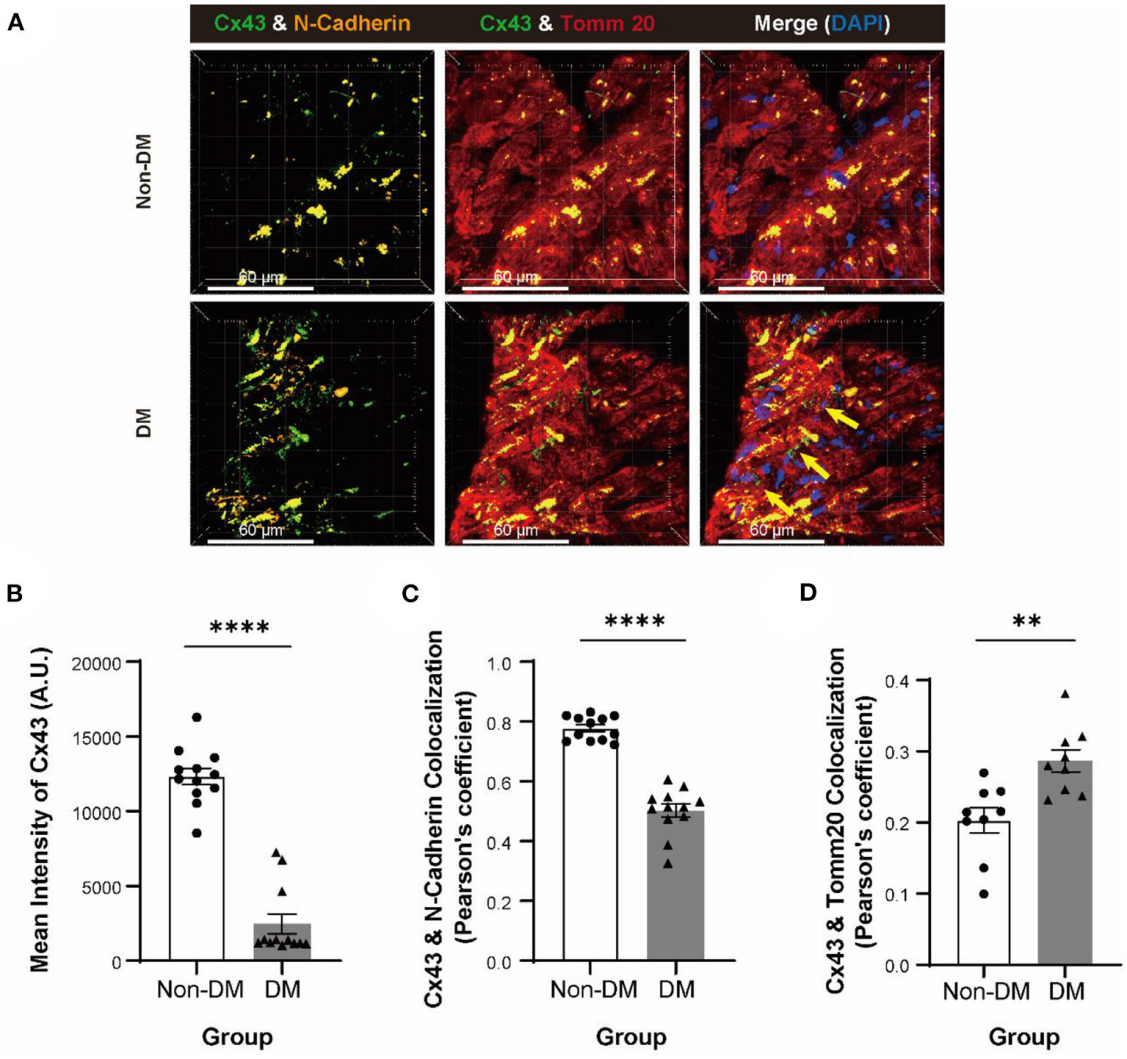
Degradation and displacement of connexin 43 (Cx43) in myocardium of patients with diabetes mellitus (DM). **(A)** Immunofluorescent staining of Cx43, N-cadherin, and Tomm20 in myocardial tissue of patients with or without DM (yellow arrow: aberrant Cx43 in mitochondria, *n* = 3 patients per group; scale bars, 60 μm). **(B–D)** Analysis of mean intensity of Cx43 [**(B)**, *n* = 12 field views per group], colocalization of Cx43 and N-cadherin [**(C)**, *n* = 12 field views per group] and colocalization of Cx43 and Tomm20 [**(D)**, *n* = 9 field views per group]. Data are shown as mean ± SEM. The Student's two-tailed *t*-test was used. **P < 0.01, ****P < 0.0001.

In order to investigate the molecular mechanism of the cardiac conduction dysfunction of patients with DM, we performed immunofluorescent staining analysis for Cx43 on cardiac sections of patients with DM and non-DM (Figure 1A). We observed a decrease of Cx43 mean fluorescence intensity in patient with DM cardiomyocytes (Figure 1B) compared to patient with non-DM. Moreover, we also found a reduced colocalization in Cx43 and N-cadherin (marking cell–cell junctions) (Figure 1C) and a significant increase in Cx43 and translocase of outer mitochondrial membrane 20 (Tomm20; marking mitochondria) colocalization (Figure 1D). Our previous study showed that Cx43 expression was decreased in H9c2 cells under HG culture, while this downregulation was more remarkable under HLG condition (29). Therefore, we reasonably speculate that patients with DM exhibit this cardiac Cx43 manifestation due to experiencing chronic hyperglycemia or acute hypoglycemic stimulation.

### Hypoglycemia Challenge Worsens Diastolic Dysfunction and Increases Cardiac Arrhythmia Susceptibility

**Figure 2:**
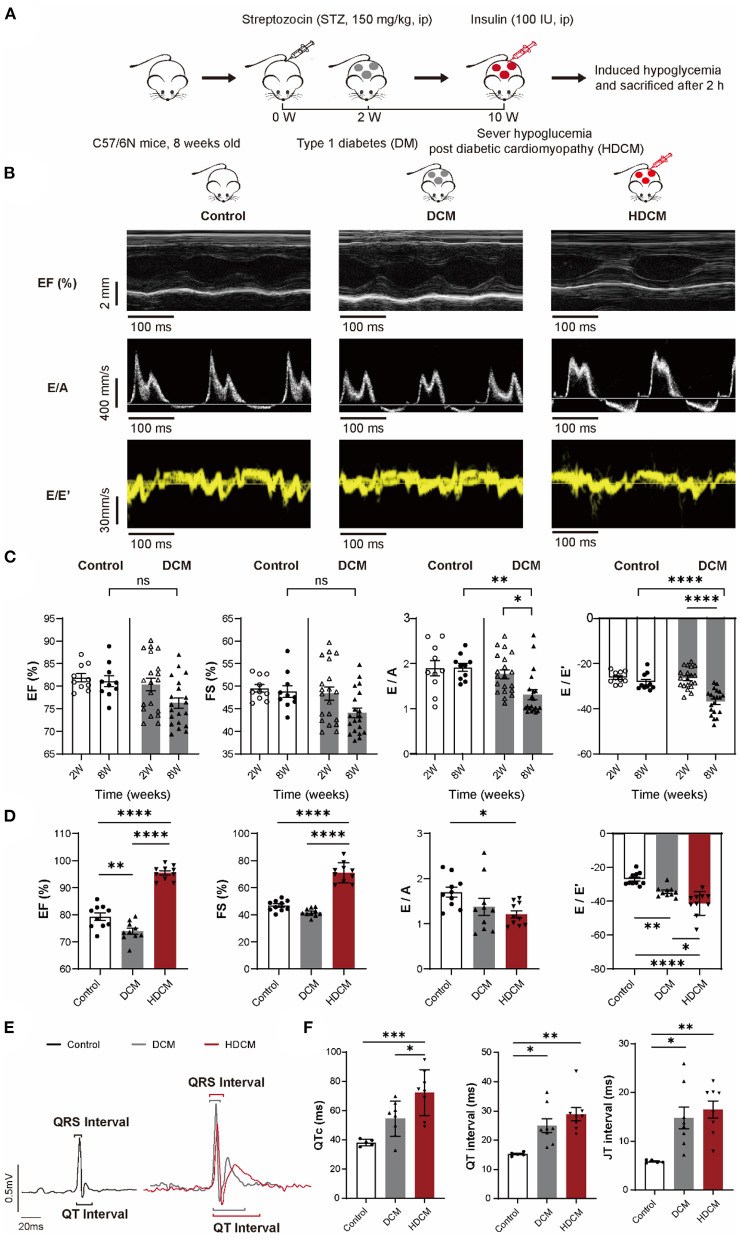
HDCM mice display worse diastolic dysfunction and increased cardiac arrhythmia susceptibility than control and diabetic cardiomyopathy (DCM) mice. **(A)** Schematic diagram of mice modeling. Mice were divided into the control group and the DM group. DM mice were injected with streptozotocin (STZ) (150 mg/kg), whereas control mice were injected with sodium citrate buffer *via* intraperitoneally injection (ip). DM mice were diagnosed with DCM via echocardiography (DCM group) at week 8. DM mice were injected with insulin (100 IU) to induce severe hypoglycemia (HDCM group) at week 10 and then sacrificed. **(B)** M model and tissue Doppler flow in control, DCM, and HDCM mice at week 10. **(C)** Echocardiography analysis of ejection fraction (EF), fractional shortening (FS), and early to late ventricular filling velocity (E/A) and early mitral inflow velocity to early diastolic mitral annulus velocity (E/E') ratios in the control (*n* = 10 mice) and DCM (*n* = 20 mice) group at both the weeks 2 and 8 post-STZ injection. **(D)** Presence of systolic and diastolic dysfunction of 10-week HDCM compared to DCM (*n* = 10 mice for per group) evaluated by echocardiography. **(E)** Representative surface ECGs of control (black, *n* = 5 mice), DCM (gray, *n* = 8 mice), and HDCM (red, *n* = 8 mice) at week 10 postinjection. **(F)** ECGs analysis of QTc, QRS interval, and QRS amplitude. Data are shown as mean ± SEM. One-way ANOVA test was used. *P < 0.05, **P < 0.01, ***P < 0.001, ****P < 0.0001.

To study the effect of hypoglycemia on cardiac function, we first induced DM (Figure 2A) in mice using the established STZ model (150 mg/kg). 2 weeks postinjection, the mice exhibited an average blood glucose level of 6.06 ± 0.30 and 29.38 ± 1.15 mM in the control and DM groups, respectively (Supplementary Figure S1A). Cardiac function was evaluated using echocardiography at 2- and 8-week post-STZ injection (Figure 2B). At 2-week post STZ injection, no significant differences in left ventricle EF, FS, E/A ratio, or E/E' ratio between DM and control mice (Figures 2B,C) were observed. At 8-week post-STZ injection, DM animals exhibited diastolic dysfunction marked by a significant decrease in E/A ratio and an increase in E/E' ratio, but no significant difference in EF and FS compared to control animals (Figure 2C), indicating that DCM disease model was successfully established. Moreover, DCM animals were significantly emaciated and depilated after 10 weeks of STZ injection (Supplementary Figures S1B,C).

We injected DCM animals with insulin to mimic hypoglycemic challenge (HDCM group; Figure 2A). 10 DCM mice were randomly selected and injected with insulin to induce hypoglycemia for 120 min prior to sacrifice. Unlike DCM and control animals, HDCM mice exhibited a significant decrease in blood glucose levels (control: 6.35 ± 0.34 mM, DCM: 26.48 ± 1.30 mM, and HDCM: 2.28 ± 0.20 mM; Supplementary Figure S1D). Moreover, HDCM animals exhibited a significant increase in EF and FS as well as a significant decrease in the E/A and E/E' ratios (Figures 2B,D), suggesting compensatory systolic function coupled with worsened diastolic function. Electrophysiologically, we observed an increase in QT interval and JT interval in DCM animals compared to control animals (Figures 2E,F) in accordance with previous observations (31). Furthermore, there was a further increase in QTc, QT interval, and JT interval in HDCM animals compared to DCM and control animals (Figures 2E,F). Together, these results suggest that hypoglycemic challenged DCM animals have worsen diastolic cardiac dysfunction and are more susceptible to ventricular arrhythmias.

### Recapitulation of Aberrant Contraction and Electrophysiology in HLG Challenged NMVMs

**Figure 3:**
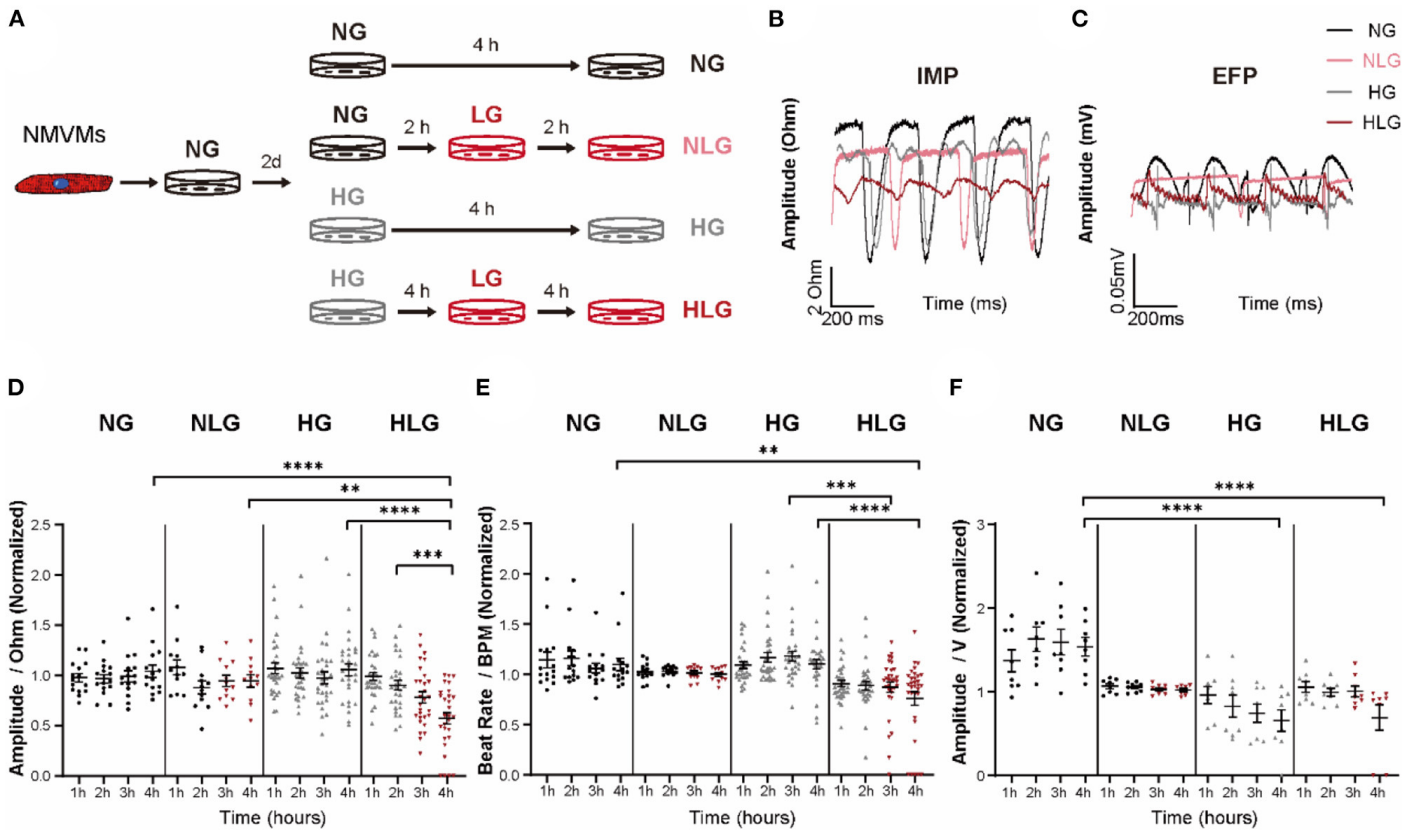
Contractility and electrophysiology disorder in cardiomyocytes from high glucose following low glucose group (HLG)-treated neonatal mouse ventricular myocytes (NMVMs). **(A)** Culture conditions and treatments of NMVMs. **(B,C)** Impedance and extracellular field potential of NMVMs after 4 h of pacing under different media conditions. **(D–F)** Analysis of impedance amplitude [**(D)**, *n* = 15 wells for normal glucose group (NG), *n* = 12 wells for normal glucose following low glucose group (NLG), *n* = 30 wells for high glucose group (HG), and *n* = 29 wells for HLG], beat rate [**(E)**, *n* = 15 wells for NG, *n* = 12 wells for NLG, *n* = 30 wells for HG, and *n* = 32 wells for HLG], and extracellular field potential [**(F)**, *n* = 8 wells per condition] changes after 4 h treatment. Data are shown as mean ± SEM. One-way ANOVA test was used. **P < 0.01, ***P < 0.001, ****P < 0.0001.

To further study the underlying mechanism that drives cardiac dysfunction upon hypoglycemic challenge, we established isolated NMVMs and treated the cardiomyocytes with either NG, NLG, HG, or HLG medium that mirrored *in-vivo* blood glucose conditions (Figure 3A). Our NMVM cultures contained ≥ 80% cardiac troponin (cTnT)^+^ cardiomyocytes evaluated by immunofluorescent staining (Supplementary Figure S2A). Next, we evaluated excitation-contraction coupling in NMVMs by measuring IMP (surrogate for contractility) and EFP (surrogate for cell surface voltage) under exogenous electrical stimulation at 1 Hz frequency (Figures 3B,C). Under continuous pacing, there was no difference in the IMP baseline among the four groups (Supplementary Figure S2B). Compared to NG, both of NLG and HG treatment did not induce any changes in IMP amplitude (Figure 3D) or beat rate (Figure 3E), but HG treatment displayed a significant decrease in EFP (Figure 3F). In the HG phase of the HLG group, we also observed no differences in IMP amplitude and beat rate (Figures 3D,E). Upon hypoglycemic challenge (media exchange at 2-h mark), we observed a significant decrease in IMP amplitude (Figure 3D), beating frequency (Figure 3E), and EFP (Figure 3F) compared to NG, NLG, and HG. These results suggest that hyperglycemia can dampen extracellular surface voltage and hypoglycemia challenge further causes the significant decrease in contraction amplitude and beat frequency of NMVMs with external electrical stimulation as well as reduced cell surface voltage. Together, we demonstrate that this NMVMs system can recapitulated our *in-vivo* and *ex-vivo* findings, which can be used for subsequent molecular characterization.

### Acute Hypoglycemia Condition Aggravates Cx43 Redistribution

**Figure 4:**
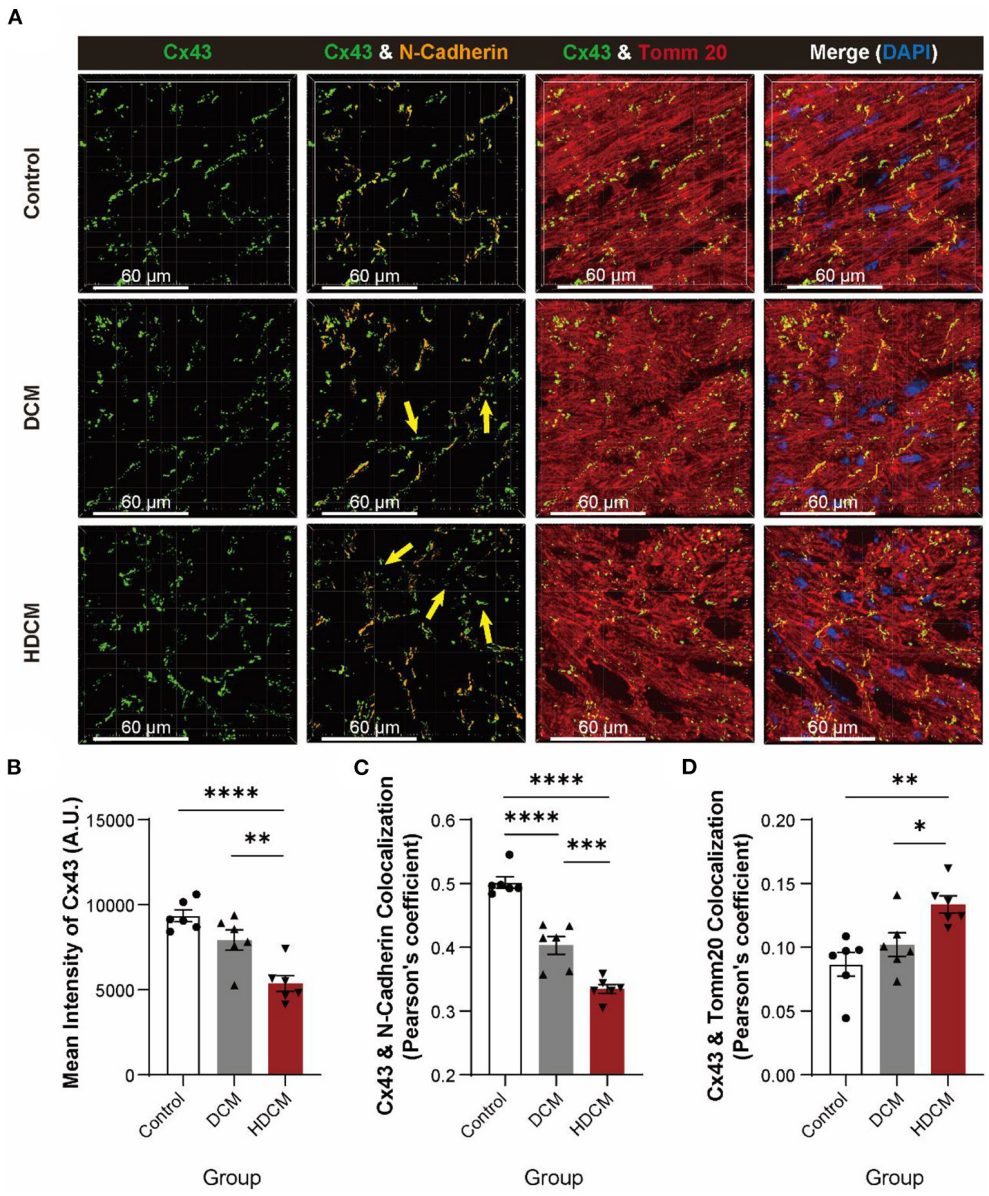
HDCM mice exhibit a worse degradation and displacement of Cx43. **(A)** Immunofluorescent staining of Cx43, N-cadherin, and Tomm20 in myocardial tissue of control, DCM, and HDCM mice (yellow arrow: aberrant Cx43 in mitochondria, *n* = 5 mice per group; scale bars, 60 μm). **(B–D)** Analysis of mean intensity of Cx43 [**(B)**, *n* = 6 field views per group], colocalization of Cx43 and N-cadherin [**(C)**, *n* = 6 field views per group], and colocalization of Cx43 and Tomm20 [**(D)**, *n* = 6 field views per group]. Data are shown as mean ± SEM. One-way ANOVA test was used. *P < 0.05, **P < 0.01, ***P < 0.001, ****P < 0.0001.

Connexin 43 has been shown to participate in synchronous calcium transient propagation in cardiomyocytes (32) and aberrant Cx43 expression and distribution have been speculated to participate in arrhythmia (32, 33). To further illustrate it, we used immunofluorescent staining to examine Cx43 protein localization in control, DCM, and HDCM murine cardiomyocytes (Figure 4A). As we found in patients with DM, we observed the same reduced mean intensity of Cx43 (Figure 4B), colocalization in Cx43 and N-cadherin (Figure 4C), and significant increase in Cx43 and Tomm20 colocalization (Figure 4D) in DCM mice, while acute hypoglycemia challenge can further intensify Cx43 translocation.

**Figure 5:**
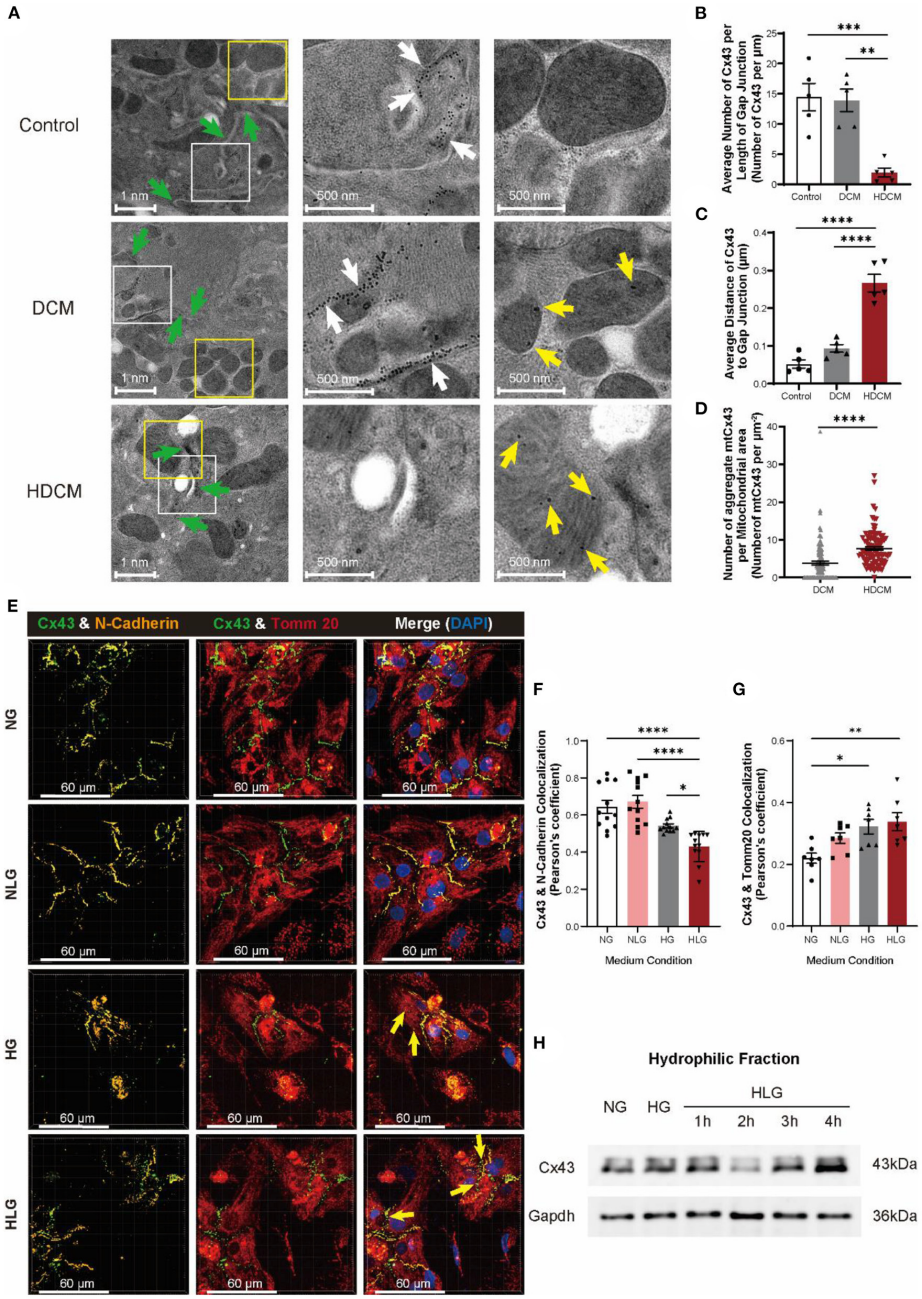
Hypoglycemia is responsible for degradation and displacement of Cx43. **(A)** Immunoelectron microscopy images of Cx43 in myocardial tissue of control, DCM, and HDCM (green arrow: cell–cell interactions; scale bars, 1 nm; white arrow: Cx43 at cell–cell interactions; scale bars, 500 nm; yellow arrow: aberrant Cx43 in mitochondria; scale bars, 500 nm, *n* = 4 mice per group). **(B)** Number of Cx43 along cell–cell interaction normalized to cell–cell interaction distance (*n* = 5 cell–cell interactions per group). **(C)** Average distance of Cx43 to cell–cell interaction (*n* = 5 cell–cell interactions per group). **(D)** Number of aggregated mtCx43 clumps averaged to mitochondria surface area (*n* = 100 mitochondria per group). **(E)** Immunofluorescent staining of Cx43, N-cadherin, and Tomm20 in NMVMs (yellow arrow: aberrant Cx43 in mitochondria; scale bars, 60 μm). **(F,G)** Analysis of colocalization of Cx43 and N-cadherin [**(F)**, *n* = 12 field views per condition], Cx43, and Tomm20 [**(G)**, *n* = 7 field views per condition]. **(H)** Western blot of Cx43 in hydrophilic cell membrane of NMVMs after hypoglycemia change. Data are shown as mean ± SEM. One-way ANOVA test was used. *P < 0.05, **P < 0.01, ***P < 0.001, ****P < 0.0001.

Next, we used immunoelectron microscopy to further examine Cx43 translocation. In control cardiomyocytes, Cx43 localized to cell–cell junctions (Figure 5A, white arrows). Interestingly, we clearly observed mtCx43 aggregation in DCM and HDCM murine cardiomyocytes, but not in controls (Figure 5A, yellow arrows). Compared to controls, DCM and HDCM murine cardiomyocytes exhibited a significant decrease in number of Cx43 located at the cell–cell junction (Figure 5B). There was a significant increase in Cx43 migratory distance away from cell membrane interface in HDCM cardiomyocytes compared to DCM and control cardiomyocytes (Figure 5C). Next, we quantified number of mtCx43 per mitochondrial cross-sectional area as a readout of mtCx43 density. We found although abnormal aggregation of mtCx43 could be observed in cardiomyocytes of both the DCM and HDCM mice and mtCx43 density is significantly greater in HDCM cardiomyocytes compared to DCM cardiomyocytes (Figure 5D). These data indicate that a certain amount of mtCx43 in DCM mice can maintain a compensatory balance, but too much mtCx43 in HDCM mice breaks this balance, resulting in a rapid decline in myocardial cell function.

To test if glucose fluctuation alone is capable of inducing Cx43 translocation in cardiomyocytes, we challenged NMVMs with varying glucose conditions and performed immunofluorescent staining for N-cadherin, Tomm20, and Cx43 (Figure 5E). Similar to our *in-vivo* observations, Cx43 and N-cadherin colocalization in NMVMs decreased significantly post 4-h HG treatment and were further exacerbated in the HLG group (Figure 5F). A similar trend was observed for the mean Cx43 fluorescence intensity (Supplementary Figures S3A,B), while no change in N-cadherin fluorescence intensity was observed (Supplementary Figure S3C). Notably, Cx43 and Tomm20 colocalization significantly increased upon HLG treatment (Figure 5G). NLG challenge resulted in an overall decrease in Cx43 protein (Supplementary Figure S3B), a decrease in membrane Cx43 (Figure 5F), yet like HG challenge, enhanced mitochondrial accumulation (Figure 5G). Furthermore, we observed a loss of membrane Cx43 by immunoblotting of the hydrophobic fraction at 2-h posthypoglycemic challenge (Figure 5H). Together, these data demonstrate that hypoglycemic challenge causes Cx43 proteins to be lost from cardiomyocyte cell–cell junctions and aggregate in mitochondria. This also supports our speculation that changes of Cx43 in myocardium of patients with DM are due to hypoglycemic challenge.

### Src-Mediated Cx43 Phosphorylation Drives Cx43 Translocation to the Mitochondria

**Figure 6:**
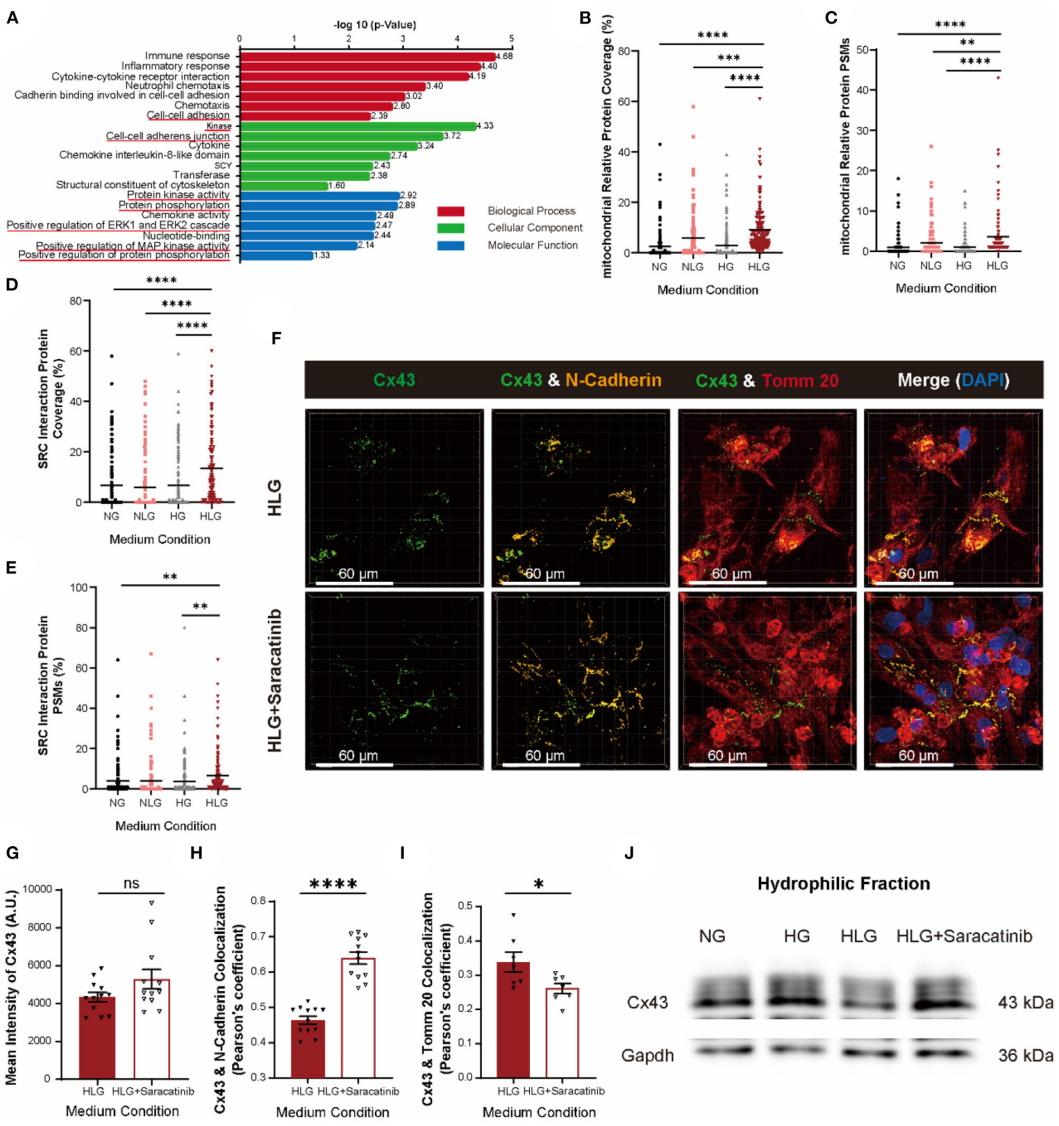
Activation of Src protein regulates the entry of Cx43 into mitochondria. **(A)** The Gene Ontology pathway analysis of activated genes under HLG treatment. **(B,C)** Coverage and propensity score matching (PSM) analysis of mitochondria relative proteins (*n* = 162 proteins per condition). **(D,E)** Coverage and PSMs analysis of Src-interacting proteins (*n* = 131 proteins per condition). Data are shown as mean ± SEM. One-way ANOVA test was used. **P < 0.01, ***P < 0.001, ****P < 0.0001. **(F)** Colocalization of Cx43 and Tomm20 in HLG and saracatinib-treated HLG NMVMs (scale bars, 60 μm). **(G–I)** Analysis of mean intensity of Cx43 [**(G)**, *n* = 12 per condition], Cx43, and N-cadherin [**(H)**, *n* = 12 per condition], and colocalization of Cx43 and Tomm20 [**(I)**, *n* = 7 per condition]. **(J)** Western blot of Cx43 in hydrophilic cell membrane of HLG and saracatinib-treated HLG NMVMs. Data are shown as mean ± SEM. The Student's two-tailed *t*-test was used. *P < 0.05, ****P < 0.0001.

To identify proteins responsible for Cx43 internalization and translocation to mitochondria upon hypoglycemia challenge, we performed Co-IP coupled with mass spectrometry (Co-IP/MS) using antibody against Cx43 protein in NMVMs cultured under the NG, NLG, HG, and HLG conditions. Proteins mapped were used for the downstream Gene Ontology enrichment analysis. Compared to those under the NG, NLG, and HG conditions, kinase activity, protein phosphorylation, cell–cell adhesion, and cytoskeleton-associated proteins were significantly increased under the HLG conditions (Figure 6A). The percentage and propensity score matching coverage of mitochondria-associated proteins bound to Cx43 increased significantly upon HLG induction (Figures 6B,C and Supplementary Figure S4A). These results are consistent with the increase in mtCx43 accumulation upon hypoglycemia induction (Supplementary Figures 5A–G). Furthermore, Src and Src-interacting proteins were enriched in our Cx43 Co-IP/MS analysis (Figures 6D,E, Supplementary Figures S4B,C). In addition, immunofluorescent staining data showed that HLG culture could significantly increase the correlation coefficient between Src and Cx43 (Supplementary Figures S4D,E). To test if Cx43 translocation was mediated by Src, we treated HLG NMVMs with saracatinib, a Src inhibitor (Figure 6F). Saracatinib did not change the overall expression of Cx43 (Figure 6G), which suggests that saracatinib does not interfere with Cx43 turnover. Moreover, saracatinib significantly increased Cx43 and N-cadherin colocalization (Figure 6H) and decreased Cx43 and Tomm20 colocalization (Figure 6I) in NMVMs cultured under the HLG conditions. Last, saracatinib restored Cx43 protein levels located at the membrane fraction (Figure 6J).

**Figure 7:**
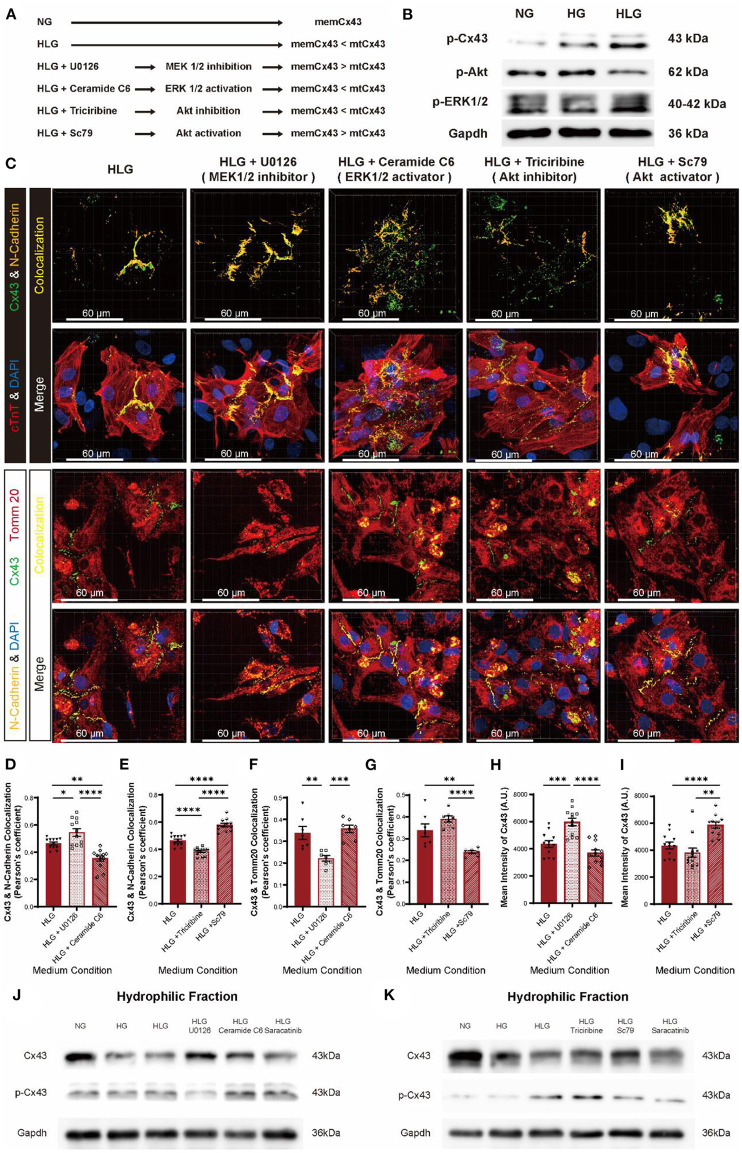
MEK/ERK and PI3K/Akt pathways regulate Cx43 transfer into mitochondria through Src. **(A)** Schematic diagram of proposed abnormal Cx43 aggregation in mitochondria mechanism. **(B)** Phosphorylation of Cx43, Akt, and ERK1/2 under HLG condition. **(C)** Immunofluorescent staining of Cx43 of the HLG group under drug stimulation (scale bars, 60 μm). **(D–I)** Analysis of colocalization of Cx43 and N-cadherin [**(D,E**), *n* = 12 field views per condition], Cx43 and Tomm20 [**(F,G)**, *n* = 7 field views per condition], and mean intensity of Cx43 [**(H,I)**, *n* = 12 field views per condition]. **(J,K)** Western blot of Cx43 and p-Cx43 in hydrophilic cell membrane of HLG with activators or inhibitors cultured NMVMs. Data are shown as mean ± SEM. One-way ANOVA test was used. *P < 0.05, **P < 0.01, ***P < 0.001, ****P < 0.0001.

Although we identified Src as the key player mediating Cx43 dissociation from cell membrane, but upstream activators remain elusive. In our Co-IP/MS, we identified mitogen-activated protein kinase 1 (MAPK1), MAP2K1, PI3K, and Akt, which belong to the MEK/ERK and PI3K/Akt pathways (Figure 6A) as potential Src-interacting partners (Figure 7A). Compared to the NG and HG groups, HLG NMVMs exhibited ERK1/2 upregulation (high p-ERK1/2 signal) and Akt downregulation (low p-Akt signal) (Figure 7B). To elucidate the regulatory role of MEK/ERK1/2 and PI3K/Akt pathways in Src-mediated Cx43 translocation, we treated NMVMs cultured under the HLG conditions with either MEK inhibitor (U0126), ERK activator (ceramide C6), Akt inhibitor (triciribine), or Akt activator (Sc79) and measured Cx43-N-cadherin and Cx43-Tomm20 colocalization. Inhibition of the MEK pathway (U0126) and activation of the Akt pathway (Sc79) significantly reduced Cx43 dissociation from cell–cell junctions (Figures 7C–E), decreased Cx43 mitochondrial aggregation (Figures 7C,F,G), and increased Cx43 fluorescence intensity (Figures 7C,H,I) in HLG NMVMs. Conversely, ERK activation (ceramide C6) or Akt inhibition (triciribine) significantly increased Cx43 dissociation from cell–cell junctions (Figures 7C–E), increased Cx43 mitochondrial aggregation (Figures 7C,F,G), and decreased Cx43 fluorescence intensity (Figures 7C,H,I) in HLG NMVMs. In isolated cell membrane fraction, HG and HLG resulted in loss of membrane Cx43 protein (Figure 7J); reciprocal increase in p-Cx43 was also observed in hydrophilic fraction (Figure 7K). By modulating MEK and Akt pathways, we detected an increase in Cx43 protein levels in HLG NMVMs treated with MEK inhibitor (U0126) or Akt activator (Sc79); ERK activator (ceramide C6) and Akt inhibitor (triciribine) treatments did the reverse action (Figures 7J,K). Together, these data suggest that HLG challenge activates MEK/ERK pathway and/or inhibits PI3K/Akt pathway to mediate Cx43 membrane dissociation and translocation to mitochondria.

**Figure 8:**
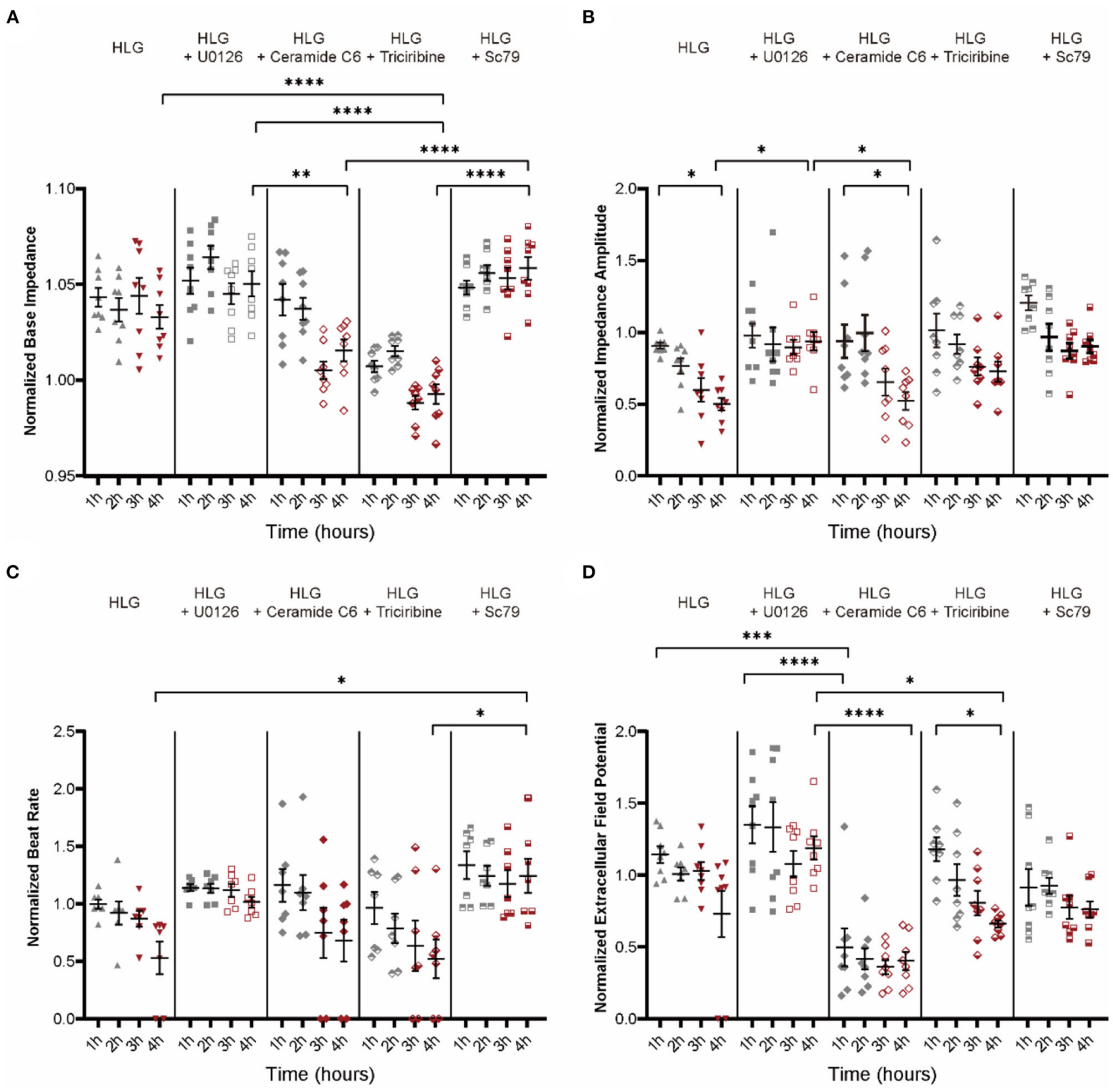
Electrophysiology function of NMVMs after treatment with activators or inhibitors of MEK/ERK and PI3K/Akt pathways. **(A–D)** Analysis of base impedance [**(A)**, *n* = 8 wells per condition], impedance amplitude [**(B)**, *n* = 8 wells per condition], beat rate [**(C)**, *n* = 7 wells per condition], and extracellular field potential changes [**(D)**, *n* = 8 wells per condition] in drug-stimulated HLG NMVMs. Data are shown as mean ± SEM. One-way ANOVA test was used. *P < 0.05, **P < 0.01, ***P < 0.001, ****P < 0.0001.

Building on the HLG NMVMs model, MEK inhibitor (U0126) and Akt activator (Sc79), which blocked Cx43 dissociation, did not alter IMP baseline; however, ERK activator (ceramide C6) and Akt inhibitor (triciribine) significantly lowered IMP baseline (Figure 8A) prior to low glucose switch. Functionally, MEK inhibition (U0126) and Akt activation (Sc79) prevented loss of IMP amplitude (Figure 8B), beat rate (Figure 8C), and EFP (Figure 8D) upon low glucose switch. Conversely, ERK activator (ceramide C6) and Akt inhibitor (triciribine) failed to confer protection upon HLG challenge (Figures 8B–D). Western blot data showed that inhibiting MEK/ERK pathway or activating PI3K/Akt pathway could inhibit HLG-induced Src activation (Supplementary Figure S4F). Together, these data demonstrate that hypoglycemic challenge activates MEK/ERK and/or inhibits PI3K/Akt pathways that converge at Src to drive Cx43 mitochondrial translocation.

### Overexpression of MtCx43 Results in Worse Cardiac Dysfunction and Risk of Arrhythmia Susceptibility

**Figure 9:**
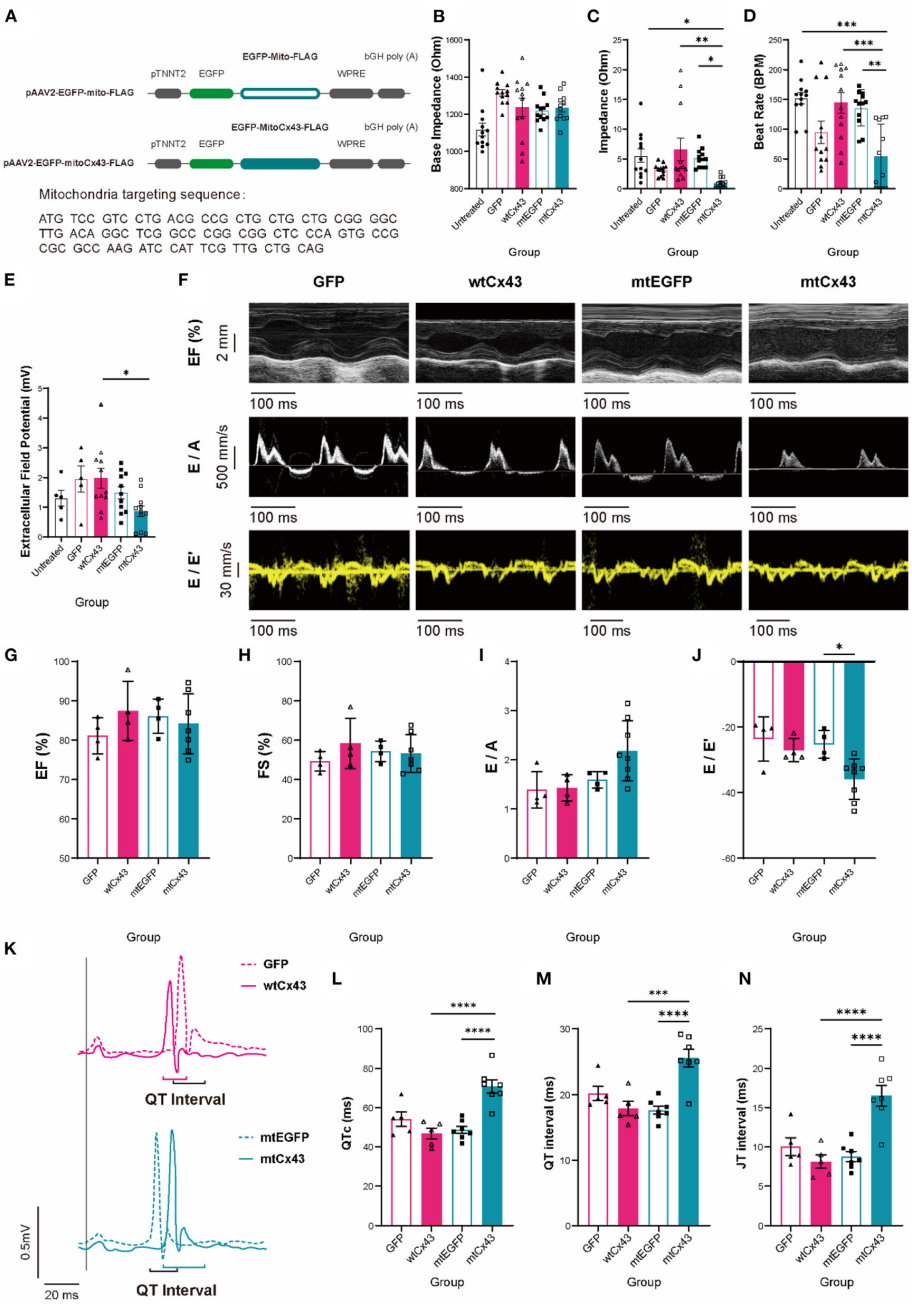
Mitochondrial Cx43 overexpression causes aberrant cardiac contraction, electrophysiological abnormalities, and increased fatal arrhythmias susceptibility. **(A)** Recombinant p-AAV vectors of mito-GFP-FLAG and mito-Cx43-FLAG. **(B–E)** Base impedance [**(B)**, *n* = 12 wells per group], amplitude [**(C)**, *n* = 12 wells for per group], beat rate [**(D)**, *n* = 12 wells for per group], and extracellular field potential changes [**(E)**, *n* = 5 wells for the untreated and GFP groups, *n* = 11 wells for the wtCx43, mtEGFP, and mtCx43 groups] of untreated (black), GFP (shaded peach pink), wtCx43 overexpression (solid peach pink), mtEGFP (shaded teal), and mtCx43 overexpression (solid teal) under electrostimulation. Data are shown as mean ± SEM. One-way ANOVA test was used. *P < 0.05, **P < 0.01, ***P < 0.001. **(F)** M-model and tissue Doppler flow in mtGFP and mtCx43 overexpression mice on week 2. **(G–J)** Echocardiography analysis of EF **(G)**, FS **(H)**, E/A **(I)**, and E/E' **(J)** (*n* = 4 mice for GFP, wild-type Cx43 (wtCx43), and the mtEGFP group, *n* = 7 mice for the mtCx43 overexpression group). Data are shown as mean ± SD. The Student's two-tailed *t*-test was used. *P < 0.05. **(K)** ECGs of GFP (shaded peach pink), wtCx43 overexpression (solid peach pink), mtEGFP (shaded teal), and mtCx43 overexpression (solid teal) group. **(L–N)** Summary of QTc **(L)**, QRS interval **(M)**, and QRS amplitude **(N)** of GFP, wtCx43, mtEGFP, and the mtCx43 overexpression group (*n* = 5 mice for GFP and the wtCx43 overexpression group, *n* = 7 mice for each of the mtGFP and mtCx43 overexpression group). Data are shown as mean ± SEM. The Student's two-tailed *t*-test was used. ***P < 0.001, ****P < 0.0001.

To determine whether mtCx43 is sufficient to increase the risk of cardiac dysfunction and susceptibility of arrhythmias, we constructed an AAV overexpression vector containing a mitochondrial localization sequence fused to either Cx43 (EGFP-mtCx43) or EGFP (mtEGFP) (Figure 9A and Supplementary Figure S5A) with wtCx43 (wtCx43-GFP) or GFP as controls. First, we confirmed that overexpression of mtCx43 in NMVMs showed an increase in the Cx43 signal at mitochondria by immunofluorescence (Supplementary Figure S5C, yellow arrows). Functionally, overexpression of mtCx43, but not wtCx43, in NMVMs, resulted in a significant increase in base IMP (Figure 9B) and decrease in IMP (Figure 9C) as well as beat rate (Figure 9D) and a reduced change in EFP (Figure 9E).

To further validate whether overexpression of mtCx43 can lead to cardiac dysfunction, wild-type animals were injected with AAV2/9 viruses overexpressing mtCx43, wtCx43, mtEGFP, or GFP and expression was confirmed by immunoelectron microscopy (Supplementary Figure S5B). Cardiac functions were evaluated by echocardiography (Figure 9F). In mtCx43 overexpression animals, we did not observe any differences in EF, FS, or the E/A ratio (Figures 9G–I) compared to wtCx43, mtEGFP, and GFP animals; however, we observed a significant decrease in the E/E' ratio in the mtCx43 overexpression group in week 2 (Figure 9J), suggestive of mild diastolic function. As expected, only mtCx43 overexpression induced QTc, QT interval, and JT interval prolongation (Figures 9K–N), suggestive of increased ventricular arrhythmia susceptibility.

In summary, we demonstrated that hypoglycemic challenge is responsible for dissociation and mitochondrial translocation of Cx43 in diabetic myocardium. Hypoglycemia stimulation worsened diastolic cardiac dysfunction and increased the risk of ventricular arrhythmia in STZ-induced DCM murine model. Cellularly and molecularly, we demonstrated that hypoglycemia activated MEK/ERK and inhibited PI3K/AKT pathways that converge on Src protein, which phosphorylates Cx43 and drives its translocation to the mitochondria. Further, by overexpressing mitochondrial localizing Cx43, we demonstrated that mtCx43 aggregation can result in contractile dysfunction at the cellular level, causes cardiac dysfunction and increases the risk of ventricular arrhythmia *in vivo*. Our results provide novel molecular insights into the pathogenesis of hypoglycemia-aggravated DCM and identify novel targets for future therapeutic designs.

## Discussion

We provide evidence for Src-driven mtCx43 responsible for DCM defects in human and murine cardiomyocytes. This is remarkable, as in absence of hypoglycemic challenge, AAV2/9-mtCx43 overexpression was capable of inducing diastolic dysfunction and arrhythmia susceptibility marked by prolonged QT intervals. In isolated adult and neonatal mouse ventricular cardiomyocytes, we show that glucose manipulation is a great way to mimic hypoglycemia events and allows the precise characterization of Cx43 localization. Mechanistically, we demonstrate that MEK/ERK and PI3K/Akt pathways are upstream of Src activation. Using pharmacological means, we showed that MEK/ERK and PI3K/Akt modulation can enhance or prohibit Cx43 translocation.

Clinically, patients with DM treated with insulin exhibited left ventricular hypertrophy and greater diastolic dysfunction compared to patients without insulin prescription—possibly due to acute hypoglycemia induced by insulin therapy (34). In keep with previous finding (35), we find that acute hypoglycemic shock greatly dampens myocardial function. The observed prolongation of QT interval and QRS interval—events that lead to ventricular arrhythmias—have been highly correlated with an increased mortality during severe hypoglycemic events (36). Similar to Cx43 remodeling that happens prior to cardiac remodeling in large animal model of nonischemic heart failure (37), we too find that mtCx43 overexpression is sufficient for inducing cardiac dysfunction in absence of cardiac fibrosis.

Connexin 43 is the most ubiquitously expressed GJ protein found in almost all the tissue types. Cx43 expression levels have been implicated in mitochondrial homeostasis (38), autophagy regulation (39), intracellular trafficking (40), and long-distance communication mediated *via* extracellular vesicles (EVs) (41). In accordance to our previous observation (29), we find that hypoglycemia activates Src via MEK/ERK and PI3K/Akt pathways, which results in mtCx43 accumulation. Interestingly, Src has been implicated in Cx43 dysregulation in the heart (42–45), but most studies attributed cardiac dysfunction to loss of cell–cell junction. Here, we demonstrate that in presence of intact cell–cell junctions and absence of hypoglycemia, overexpression of mtCx43 induced diastolic dysfunction and increase the risk of arrhythmia susceptibility. Apart from being used to treat cancer (46), Src inhibitor saracatinib (AZD0530) has been shown to confer ~50% improvement in cardiac conduction velocity and lowered overall arrhythmia episodes by stabilizing membrane Cx43 (43). Based on our results, we would argue that the efficacy of saracatinib is less likely due to the increase of membrane Cx43, but rather the prevention of mtCx43 accumulation.

Our MS data also proves that Cx43 binds to IMM (38), yet the role and function of mtCx43 remains controversial (47–51). The observed large Cx43 nanogold aggregates are suggestive to the formation of mtCx43 hemichannels. MtCx43 hemichannels could potentially act as large conductance channels that dissipate mitochondrial membrane potential and disrupt ATP synthesis (38); in open states, Cx43 hemichannels could elicit myocardial death (50). In addition to metabolic homeostasis, impaired mitochondria Ca^2+^ handling plays a key role in the development of the cardiac diastolic dysfunction characteristic of early DCM (2). Although the percentage of mtCx43 hemichannels in patients with DM remains to be determined, disruption of mitochondrial membrane potential and induction of aberrant Ca^2+^ handling align with DCM progression—a notion which our *in-vivo* and *in-vitro* mtCx43 overexpression results support.

In summary, this study provides mechanistic insights by which mtCx43 translocation aggravates DCM. Based on our findings, we boldly speculate that chronic mtCx43 accumulation may be compensatory adaptation of DCM, yet acute hypoglycemic challenges may lead to Cx43 hemichannel opening and disrupt cardiac electrophysiology, aggravate DCM in murine STZ model as well as NMVMs. Restoration of membrane Cx43 and prevention of mtCx43 accumulation offer a new therapeutic possibility for the prevention of sudden cardiac death and DCM progression.

## Data Availability Statement

The raw data supporting the conclusions of this article will be made available by the authors, without undue reservation.

## Ethics Statement

The studies involving human participants were reviewed and approved by the Ethics Review Committee at Ninth People's Hospital, Shanghai Jiao Tong University School of Medicine, China. The patients/participants provided their written informed consent to participate in this study. The animal study was reviewed and approved by the Laboratory Animal Care Ethics Review Committee at the Ninth People's Hospital, Shanghai Jiao Tong University School of Medicine, China. Written informed consent was obtained from the individual(s) for the publication of any potentially identifiable images or data included in this article.

## Author Contributions

XW, ACYC, and QZ conceived the study. XW carried out the experiments and the data analysis. ML, ACYC, and QZ contributed to the design of the experiments, supervised analysis, and interpretation. ACHC conducted experiments and modified the language of the article. HC and SX contributed to sample preparation and immunoelectron microscopy. YX and YZ helped in data analysis of immunofluorescence staining images and immunoelectron microscopy images. XW wrote the manuscript in consultation with ACYC and QZ who supervised the project. All authors discussed the results and contributed to the final manuscript.

## Funding

This study was supported by the National Natural Science Foundation of China (Grant Numbers 81771496 to QZ and 82070248 to ACYC), the Shanghai Pujiang Program (Grant Number 19PJ1407000 to ACYC), the Program for Professor of Special Appointment (Eastern Scholar) at Shanghai Institutions of Higher Learning (Grant Number 0900000024 to ACYC), and the Innovative Research Team of High-Level Local Universities in Shanghai (Grant Number SHSMU-ZLCX20211700 to ACYC).

## Conflict of Interest

The authors declare that the research was conducted in the absence of any commercial or financial relationships that could be construed as a potential conflict of interest.

## Publisher's Note

All claims expressed in this article are solely those of the authors and do not necessarily represent those of their affiliated organizations, or those of the publisher, the editors and the reviewers. Any product that may be evaluated in this article, or claim that may be made by its manufacturer, is not guaranteed or endorsed by the publisher.
