# Supplementary material for: Hypoglycemia-Exacerbated Mitochondrial Connexin 43 Accumulation Aggravates Cardiac Dysfunction in Diabetic Cardiomyopathy
Source: Front Cardiovasc Med. 2022 Mar 16;9:800185. doi: 10.3389/fcvm.2022.800185 (PMC8967291; doi:10.3389/fcvm.2022.800185)
Supplement: Supplementary file 3 [file Data_Sheet_3.pdf]

Supplementary Tables and Figure Legends

TABLE S1: General clinical characteristic of Non-DM and DM patients.

FIGURE S1: Changes in blood glucose levels, body weight and body size of experimental animals. (A) Blood glucose levels in experimental animals post STZ or saline injection (measured in the morning) (n = 10 mice for Control group, n = 20 mice for DM group). (B) Body weight changes in experimental animals post STZ or saline injection (weighed in the morning) (n = 5 mice per group). (C) Comparison of mice in Control and DCM 10 weeks after injection. (D) Blood glucose level changes of HDCM animals over 2 h after insulin injection (n = 10 mice per group). Data are shown as mean ± SEM. One-way analysis of variance (ANOVA) was used. $^{****}P<0.0001$.

FIGURE S2: NMVMs and their beating condition. (A) NMVMs identification *via* immunofluorescence staining (Scale bars, 50 μm). (B) Analysis of base impedance changes under different culture conditions over 4 h (n = 17 wells for NG, n = 32 wells for HG, n = 31 wells for HLG). Data are shown as mean ± SEM. One-way analysis of variance (ANOVA) was used.

FIGURE S3: Expression of Cx43 and N-cadherinin after HLG treatment. (A) Immunofluorescence staining of Cx43 and N-cadherin colocalization in NMVMs (white arrow: Cx43 at cell-cell interactions, yellow arrow: Cx43 at mitochondria. Scale bars, 60μm). (B-C) Analysis of mean intensity of Cx43 and N-cadherin (n = 12 field views per condition). Data are shown as mean ± SEM. One-way analysis of variance (ANOVA) was used. $^{*}P<0.05$, $^{****}P<0.0001$.

FIGURE S4: Regulation of Src, MEK/ERK and PI3k/Akt pathways on NMVMs after HLG treatment. (A-B) Coverage changes of mitochondrial proteins and Src interactive proteins binding to purified Cx43 protein in different culture media analyzed by mass spectrometry (n = 50 proteins per condition). (C) Coverage and PSMs changes of Src binding to purified Cx43 protein in different culture conditions. (D) Immunofluorescence staining of Cx43 and Src in NMVMs (Scale bars, 60μm). (E) Analysis of colocalization of Cx43 and Src (n = 5 field views per condition). (F) Western blot of Src in differentially treated NMVMs. Data are shown as mean ± SEM. One-way analysis of variance (ANOVA) was used. $^{*}P<0.05$, $^{**}P<0.01$.

FIGURE S5: Validation of mtCx43 Overexpression *in vitro* and *in vivo*. (A) mito-Cx43-FLAG plasmid. (B) Immunofluorescence staining of Cx43, N-Cadherin and Tomm 20 in NMVMs (yellow arrows: Cx43 in mitochondria; scale bars, 60μm). (C) Mean contraction velocity of NMVMs of Untreated (black), mtEGFP (shaded teal), and mtCx43 overexpression (solid teal). (D-E) Average speed and beat rate of Untreated (black), mtEGFP (shaded teal), and mtCx43 overexpression (solid teal) (n = 10 field views per group). (F) Immunoelectron Microscopy images of Cx43 in myocardium of

Untreated, mtEGFP, and mtCx43 overexpressing mice (yellow arrows: Cx43 at mitochondria; scale bars, 500nm). Data are shown as mean ± SEM. One-way analysis of variance (ANOVA) was used. $^{**}P<0.01$, $^{***}P<0.001$, $^{****}P<0.0001$.

Supplementary Tables and Figures

TABLE S1

ID	Age	Sex	Diagnosis	Sampling location
**Control 1**	76	M	Coronary heart disease	Left auricle
**Control 2**	51	M	Aneurysm of ascending aorta, pericardium hydrops	Left auricle
**Control 3**	63	M	Severe aortic stenosis, coronary heart disease, valvular heart disease	Left auricle
**Patient 1**	77	M	Coronary heart disease, Unstable angina pectoris, essential hypertension grade III, type 2 diabetes	Left auricle
**Patient 2**	71	M	Severe aortic stenosis, hypertension, coronary heart disease, type 2 diabetes	Left auricle
**Patient 3**	67	M	Dilated cardiomyopathy, essential hypertension grade I, atrial fibrillation, type 2 diabetes	Left auricle

FIGURE S1

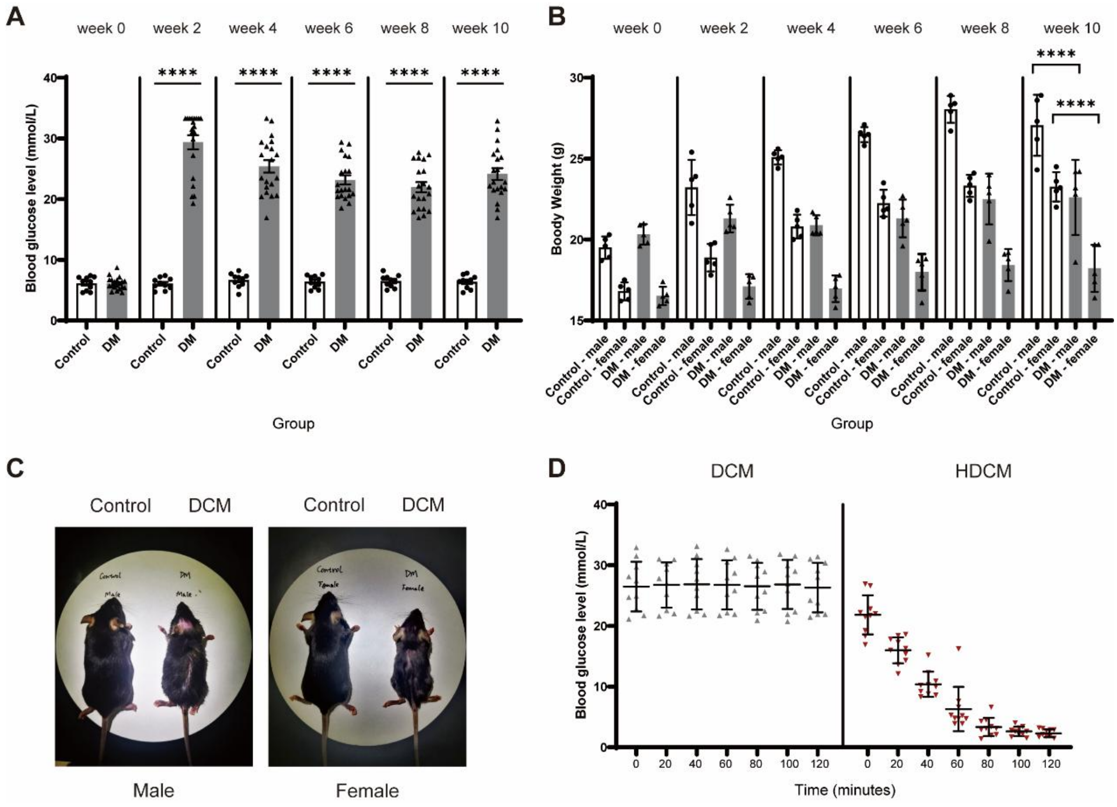
A
week 0
week 2
week 4
week 6
week 8
week 10
****
Blood glucose level (mmol/L)
Control
DM
Group
B
Body Weight (g)
Control - male
Control - female
DM - male
DM - female
C
Control
DCM
Male
Female
D
DCM
HDCM
Blood glucose level (mmol/L)
Time (minutes)

FIGURE S2

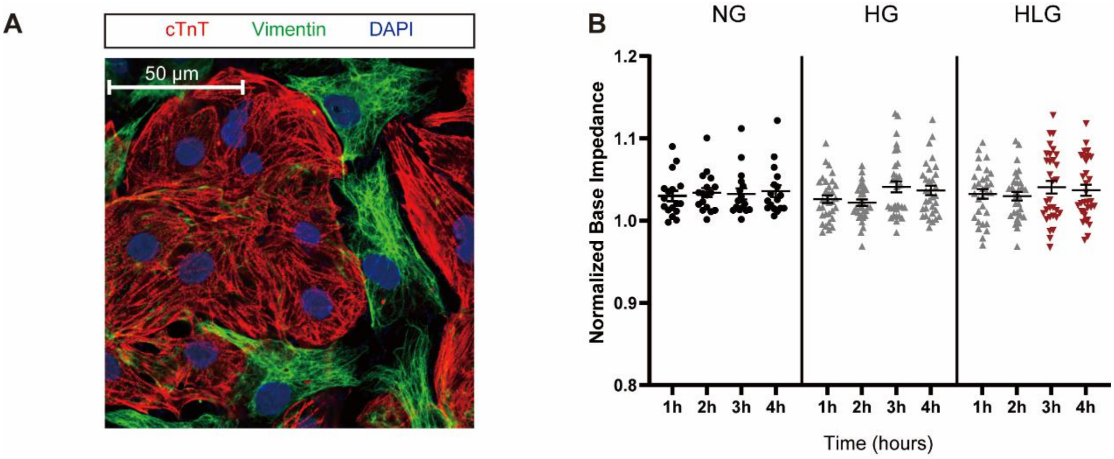
A
cTnT
Vimentin
DAPI
50 µm
B
NG
HG
HLG
Normalized Base Impedance
1.2
1.1
1.0
0.9
0.8
1h
2h
3h
4h
1h
2h
3h
4h
1h
2h
3h
4h
Time (hours)

FIGURE S3

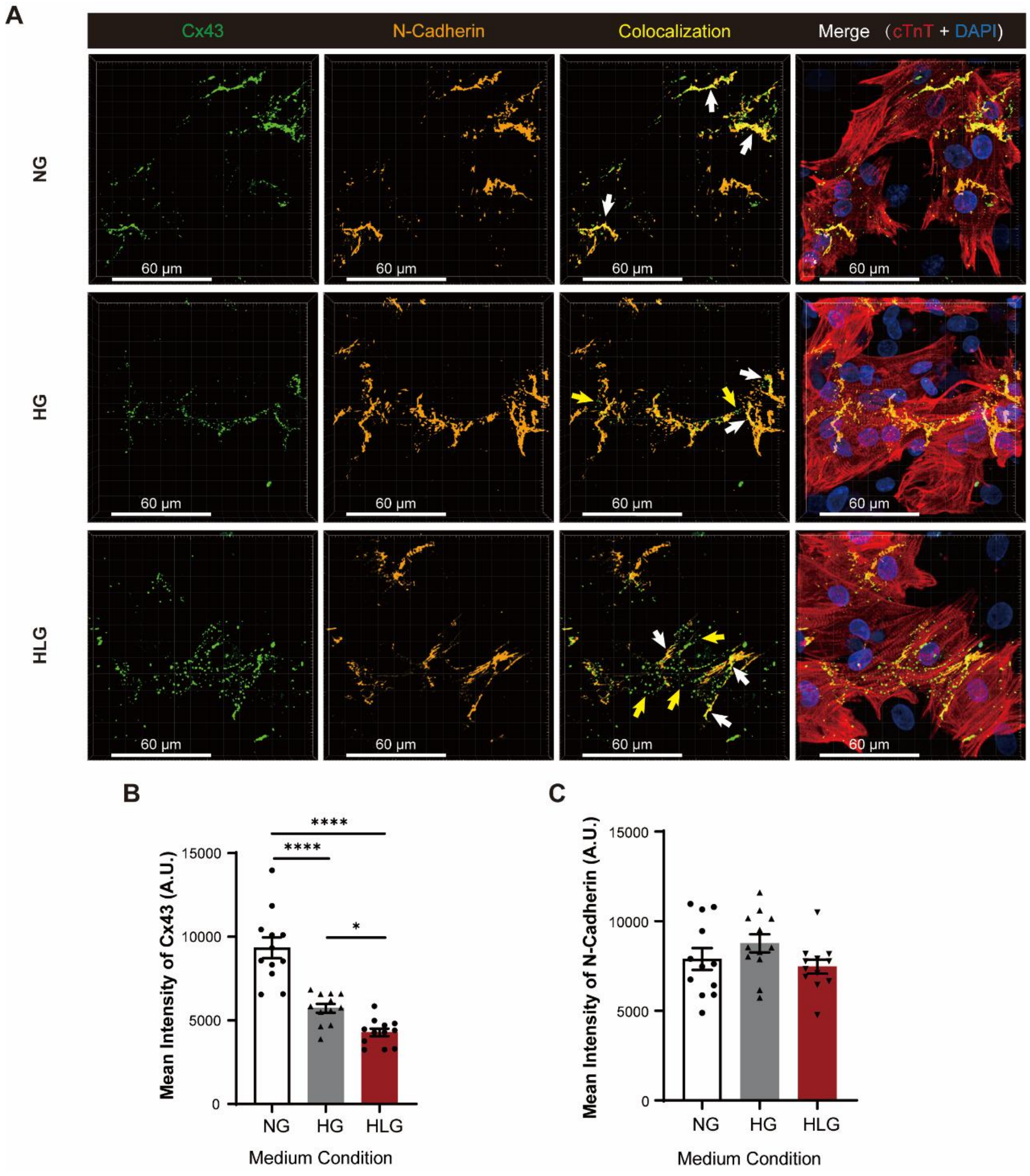
A
Cx43
N-Cadherin
Colocalization
Merge (cTnT + DAPI)
NG
HG
HLG
60 µm
B
Mean Intensity of Cx43 (A.U.)
15000
10000
5000
0
****
****
*
NG
HG
HLG
Medium Condition
C
Mean Intensity of N-Cadherin (A.U.)
15000
10000
5000
0
NG
HG
HLG
Medium Condition

FIGURE S4

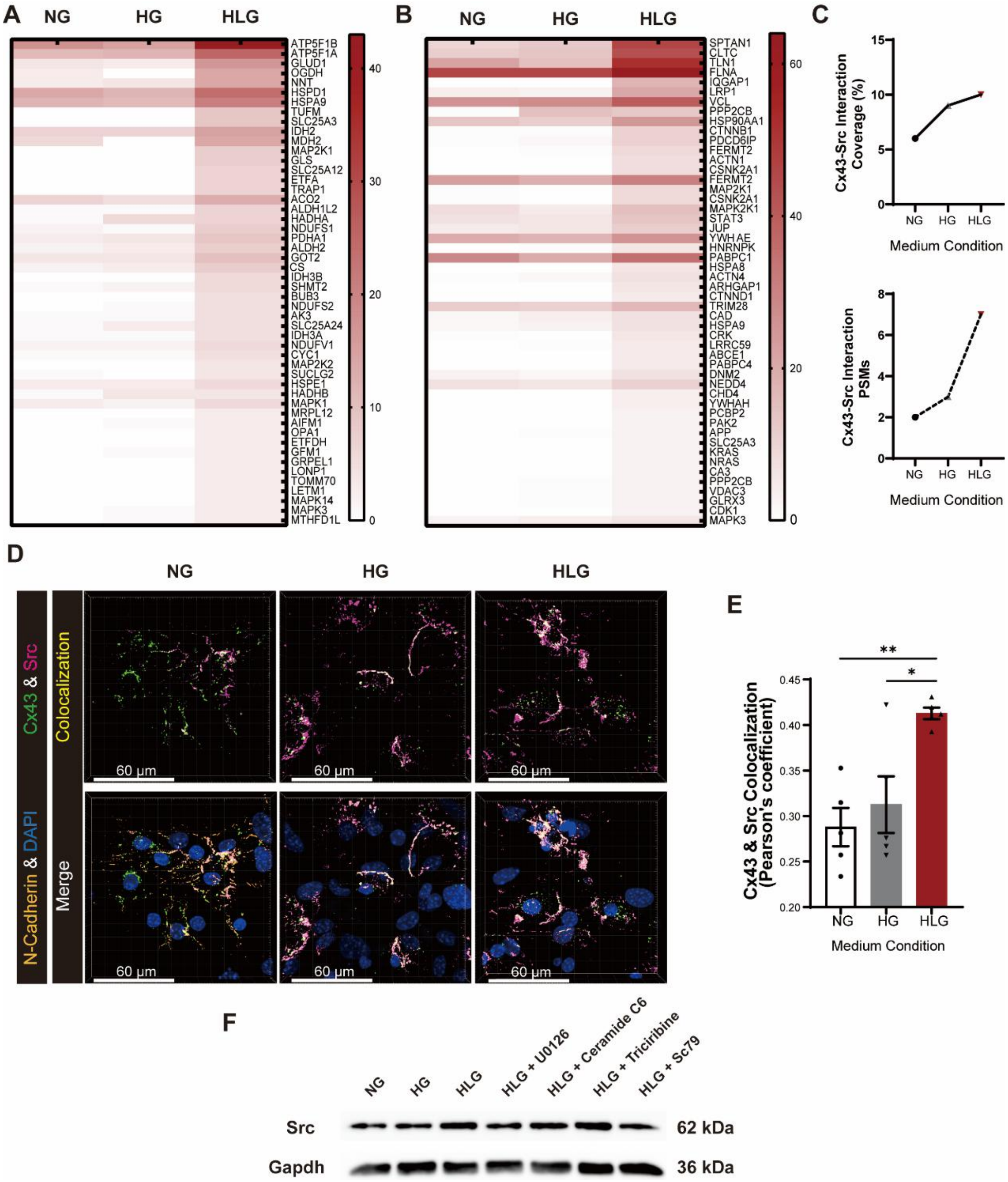
A
NG
HG
HLG
ATP5F1B
ATP5F1A
GLUD1
OGDH
NNT
HSPD1
HSPA9
TUFM
SLC25A3
IDH2
MDH2
MAP2K1
GLS
SLC25A12
ETFA
TRAP1
ACO2
ALDH1L2
HADHA
NDUFS1
PDHA1
ALDH2
GOT2
CS
IDH3B
SHMT2
BUB3
NDUFS2
AK3
SLC25A24
IDH3A
NDUFV1
CYC1
MAP2K2
SUCLG2
HSPE1
HADHB
MAPK1
MRPL12
AIFM1
OPA1
ETFDH
GFM1
GRPEL1
LONP1
TOMM70
LETM1
MAPK14
MAPK3
MTHFD1L
B
SPTAN1
CLTC
TLN1
FLNA
IQGAP1
LRP1
VCL
PPP2CB
HSP90AA1
CTNNB1
PDCD6IP
FERMT2
ACTN1
CSNK2A1
FERMT2
MAP2K1
CSNK2A1
MAPK2K1
STAT3
JUP
YWHAE
HNRNPK
PABPC1
HSPA8
ACTN4
ARHGAP1
CTNND1
TRIM28
CAD
HSPA9
CRK
LRRC59
ABCE1
PABPC4
DNM2
NEDD4
CHD4
YWHAH
PCBP2
PAK2
APP
SLC25A3
KRAS
NRAS
CA3
PPP2CB
VDAC3
GLRX3
CDK1
MAPK3
C
Cx43-Src Interaction Coverage (%)
Medium Condition
Cx43-Src Interaction PSMs
Medium Condition
D
Cx43 & Src
Colocalization
N-Cadherin & DAPI
Merge
60 μm
E
Cx43 & Src Colocalization (Pearson's coefficient)
Medium Condition
F
NG
HG
HLG
HLG + U0126
HLG + Ceramide C6
HLG + Triciribine
HLG + Sc79
Src
62 kDa
Gapdh
36 kDa

FIGURE S5

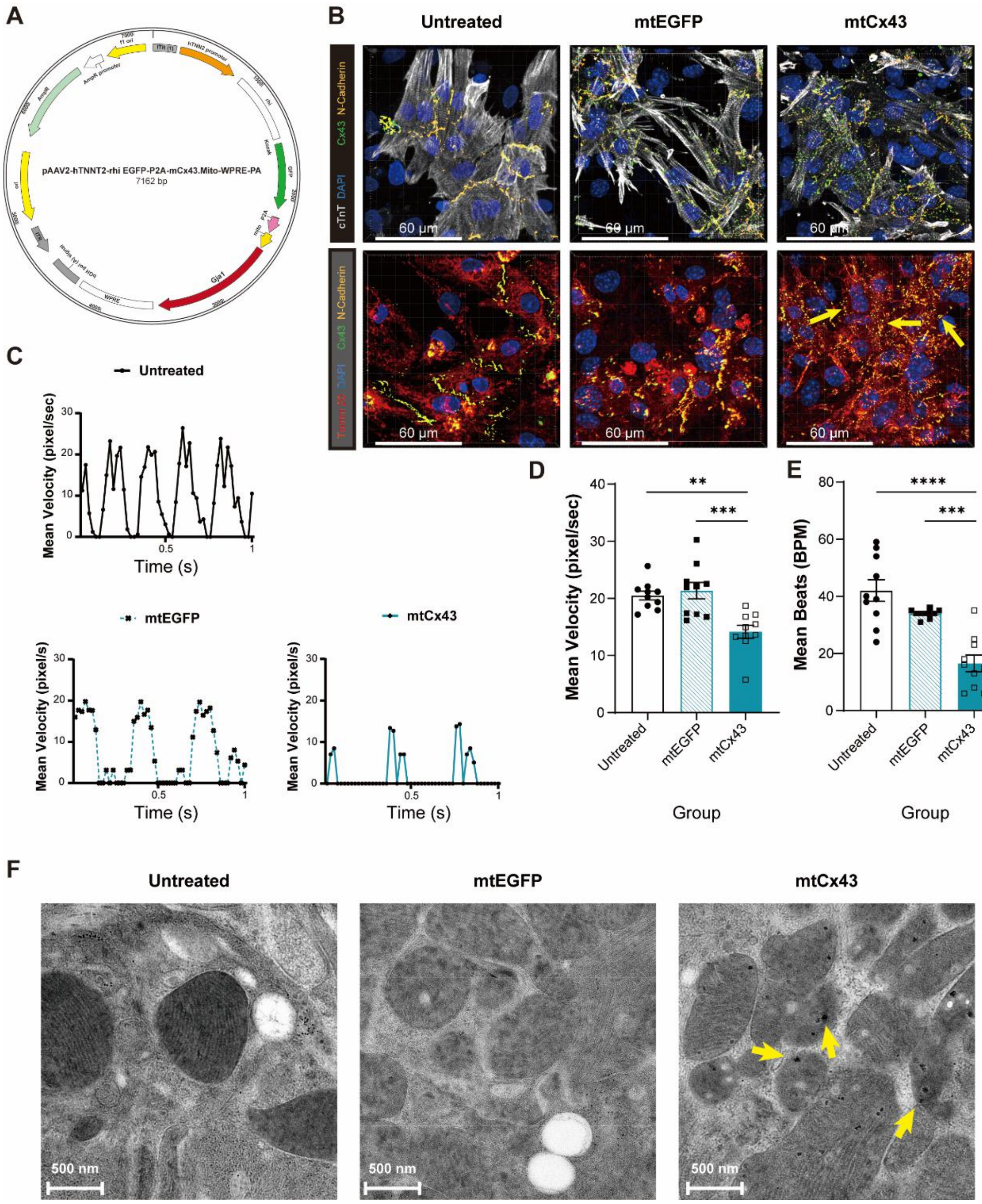
A
pAAV2-hTNNT2-rhi EGFP-P2A-mCx43.Mito-WPRE-PA
7162 bp
B
Untreated
mtEGFP
mtCx43
cTnT DAPI Cx43 N-Cadherin
Tomm20 DAPI Cx43 N-Cadherin
60 μm
C
Untreated
Mean Velocity (pixel/sec)
Time (s)
mtEGFP
mtCx43
Mean Velocity (pixel/s)
D
Mean Velocity (pixel/sec)
**
***
Untreated
mtEGFP
mtCx43
Group
E
Mean Beats (BPM)
****
***
Group
F
Untreated
mtEGFP
mtCx43
500 nm
